# Social connection and its prospective association with adolescent internalising and externalising symptoms: an exploratory cross‐country study using retrospective harmonisation

**DOI:** 10.1111/jcpp.14080

**Published:** 2024-12-07

**Authors:** Bettina Moltrecht, João Villanova do Amaral, Giovanni Abrahão Salum, Euripedes Constantino Miguel, Luis Augusto Rohde, George B. Ploubidis, Eoin McElroy, Mauricio Scopel Hoffmann

**Affiliations:** ^1^ Centre for Longitudinal Studies University College London London UK; ^2^ Graduate Program in Psychiatry and Behavioral Sciences Universidade Federal do Rio Grande do Sul Porto Alegre Brazil; ^3^ Mental Health Epidemiology Group (MHEG) Universidade Federal de Santa Maria Santa Maria RS Brazil; ^4^ Child Mind Institute New York NY USA; ^5^ Department of Psychiatry and Legal Medicine Universidade Federal do Rio Grande do Sul Porto Alegre Brazil; ^6^ National Institute of Developmental Psychiatry for Children and Adolescents (INCT‐CNPq) São Paulo Brazil; ^7^ Universidade de São Paulo São Paulo Brazil; ^8^ School of Psychology Ulster University Coleraine UK; ^9^ Department of Neuropsychiatry Universidade Federal de Santa Maria (UFSM) Santa Maria Brazil; ^10^ Care Policy and Evaluation Centre London School of Economics and Political Science London UK

**Keywords:** Adolescent mental health, social connection, country comparison, harmonisation

## Abstract

**Background:**

Social connection factors play a key role for young people's mental health. It is important to understand how their influence may vary across contexts. We investigated structural (e.g. household size), functional (e.g. social support) and quality (e.g. feeling close) social connection factors in relation to adolescent internalising and externalising symptoms, comparing two countries Brazil and the United Kingdom (UK).

**Methods:**

We pooled data from the UK Millennium Cohort Study (MCS) and the Brazilian High Risk Cohort Study (BHRCS). We included 12 social connection variables, identified through retrospective harmonisation and lived experience expert involvement. We tested measurement invariance and conducted multiple regressions to analyse associations between the social connection factors (age 14) and later internalising and externalising difficulties (age 17.5) in both cohorts. We investigated country‐level interactions and used weights to account for attrition, survey design, population representativeness and sample size.

**Results:**

We found pooled main associations with later internalising symptoms for ‘living with half‐siblings’ (*p* < .001), ‘moving address’ (*p* = .001), ‘mother marital status’ (*p* < .001–.003), ‘bullying’ (*p* = .001), ‘being bullied’ (*p* < .001) and ‘difficulties keeping friends’ (*p* < .001). For externalising, we found main associations with ‘household size’ (*p =* .041), ‘moving address’ (*p* = .041), ‘mother's marital status’ (*p* = .001–.013), ‘bullying others’ (*p* < .001) and ‘being bullied’ (*p* < .001). Country‐level interactions suggested higher internalising symptoms were associated with ‘household size’ (*p* = .001) in Brazil and ‘being bullied’ (*p* < .001) in MCS. Additionally, ‘half‐siblings in household’ (*p* = .003), ‘poor mother–child relationship’ (*p* = .018), ‘single mother’ (*p* = .035), ‘bullying’ (*p* < .001) and ‘being bullied’ (*p* < .001) were more strongly linked to externalising difficulties in MCS.

**Conclusions:**

Social connection factors, mostly structural, contributed to adolescent internalising and externalising difficulties in both countries. Factors relating to bullying and family composition seem to play a stronger role in each country. Cultural and socioeconomic factors might explain these differences. Future research should investigate cross‐regional differences to meaningfully inform global mental health efforts.

## Introduction

Mental health problems in children and adolescents are a growing global public health concern (Kieling et al., 2024). Evidence suggests that the prevalence of common psychological disorders has increased significantly over the past decades (Bor, Dean, Najman, & Hayatbakhsh, 2014; McElroy, Tibber, Fearon, Patalay, & Ploubidis, 2023), and this increase may have been further fuelled by the COVID‐19 pandemic (Bevilacqua et al., 2023). It is therefore crucial to identify active ingredients to develop and implement interventions that effectively target relevant risk and protective factors to prevent and reduce symptoms of mental ill‐health in young people (Wellcome, 2021).

Social connection – ‘a multifactorial construct that includes structural, functional, and qualitative aspects of social relationships’ and ‘encompasses the variety of ways one can connect to others socially’ (Holt‐Lunstad, Robles, & Sbarra, 2017, p. 518) – has been shown to play a key role in the mental health and well‐being of children, young people and adults alike. Social connection and its many factors have been researched for decades, and recent conceptualisation efforts have categorised the factors into three overarching domains, including a *structural* (e.g. household size, marital status, social network size), *functional* (e.g. perceived social support, perceived loneliness) and *quality* (e.g. subjective ratings of satisfaction, conflict or cohesion) domain (Holt‐Lunstad, 2021; Holt‐Lunstad et al., 2017).

Research has shown that certain factors, such as loneliness, social isolation or inter‐personal conflict in the family, school or neighbourhood environment have been associated with both greater mental and physical ill‐health (Gaertner, Fite, & Colder, 2010; Humenny, Grygiel, Dolata, & Świtaj, 2021; Kawachi & Berkman, 2001; La Greca & Harrison, 2005; Lasgaard, Goossens, Bramsen, Trillingsgaard, & Elklit, 2011; Lay‐Yee et al., 2023). Similarly, positive factors of social connection such as perceived social support seem to protect young people's mental health and well‐being (Mclaughlin & Clarke, 2010). The positive and negative impacts of different social connection factors during childhood have also been suggested to have long‐lasting effects until adulthood. Evidence from a New Zealand cohort study demonstrated that social connectedness in adolescence was a better predictor of adult well‐being than academic achievement (Olsson, McGee, Nada‐Raja, & Williams, 2013). Loneliness in childhood and adolescence on the other hand has been associated with increased risk of disability and unemployment in adulthood (von Soest, Luhmann, & Gerstorf, 2020) with earlier experiences of social adversity setting the path for increased levels of loneliness throughout life (Ejlskov, Boggild, Kuh, & Stafford, 2020).

It is not only important to identify which risk factors are linked to adolescent mental health outcomes, we must also investigate whether and how these factors differ across contexts and populations to ensure that interventions can be as effective as possible. To this day most research has been conducted in high‐income countries (Fazel, Patel, Thomas, & Tol, 2014; Patel et al., 2007), even though the majority of young people live in middle‐ and low‐income countries (Kieling et al., 2011). This evidence gap in prevention and treatment research continues to contribute to global mental health inequality. Even when data exist for different contexts, very few studies apply data harmonisation or pooling methods to conduct cross‐country research to better understand which factors are universal and which are context specific.

It can be expected that the various aspects of social connection relate differently to mental health outcomes and that they vary across contexts, as they are shaped by other societal, economic and cultural factors. Research conducted in England and Germany alone demonstrated that levels of perceived loneliness, a functional social connection factor, differed across geographical areas and related to certain characteristics in these areas, such as access to parks and leisure facilities (Buecker, Ebert, Götz, Entringer, & Luhmann, 2021; Marquez et al., 2023; Victor & Pikhartova, 2020). Two recent studies (Labella et al., 2024; Man, Liu, & Xue, 2022) investigated the prevalence of bullying and different forms of it (e.g. physical, verbal, neglect, social exclusion) across multiple countries (Man et al. = 65 and Labella et al. = 7 countries), which confirmed that bullying was present in all countries and associated with worse mental health outcomes. However, their research also provided evidence for country‐specific differences regarding the prevalence of different types of bullying, whereby social exclusion was twice as common in England than in Uruguay. Large cross‐country comparison studies like the one by Man et al. (2022) and Labella et al. (2024) are scarce, and even fewer studies have included a longitudinal design (Labella et al., 2024) to help us understand how different social risk factors influence mental health outcomes over time.

One cost‐efficient way to address this evidence gap is to utilise data from existing studies (Fortier, Doiron, Burton, & Raina, 2011). Synthesising data from multiple studies has many recognised benefits, including greater statistical power and enhanced generalizability (Fortier et al., 2017). However, data collected by individual studies tend to vary a lot even when they aim to assess similar constructs like depression or bullying. Hence, researchers need to ensure that the available data are sufficiently compatible and comparable. Retrospective measurement harmonisation is one key step in this process, whereby researchers compare and harmonise the measurement instruments (e.g. questionnaires) used in the respective studies. Detailed guidelines and reports have been published in recent years to establish a standardised approach (Fortier et al., 2017; McElory et al., 2020). For this research, we used retrospective measurement harmonisation to combine data from two existing cohort studies from the United Kingdom (UK) a high‐income country and Brazil a middle‐income country, with participants in both cohorts being born at approximately the same time (early 2000s).

Doing so allowed us to compare the two countries and examine whether social connection factors have differential associations with later adolescent mental ill‐health.

We address the following research questions:

- Which factors of social connection are associated with subsequent internalising and externalising difficulties in adolescence?
- Are there cross‐country differences for certain social connection factors and their association with later internalising and externalising difficulties in adolescence?

## Methods

### Lived experience involvement

We conducted two online workshops with young lived experience experts (LEEs), one in the UK and one in Brazil. LEEs from the UK were part of the young advisory group of the National Children's Bureau. LEEs in Brazil were active service users who were asked by their clinician if they were happy to be involved. All LEEs were compensated for their time. The workshops started with ice‐breaker activities. During the workshops, we explored with LEEs when, how and with who they experience social connection. Subsequently, we discussed how different relationships and social interactions in their daily lives influenced their mental health and well‐being. In the second part, we presented LEEs with a list of items and concepts of variables relating to social connection that are available in the UK and Brazil studies and asked them how they may relate to young people's mental health experiences. The variables had been chosen by the research team prior to the workshop using the multi‐context, multi‐domain loneliness framework (Mansfield, Henderson, Richards, Ploubidis, & Patalay, 2023). Based on feedback from LEEs, we added a new variable ‘moving house or address’ to our list of social connection factors. Furthermore, LEEs shared with us that qualitative characteristics of social relationships and interactions (e.g. do I feel judged?) may have stronger links to mental health outcomes than quantitative aspects (e.g. how many friends or siblings do I have?). The existing data from the two studies do not sufficiently cover such qualitative aspects (examples of the workshop activities and results are in the Supporting Information).

### Participants and data

Data were drawn from two longitudinal population studies – the UK‐based Millennium Cohort Study (MCS) and the Brazilian High Risk Cohort Study (BHRCS) for Childhood Mental Health Conditions. MCS has been following a representative sample of 19,000 children from all four nations of the UK born between 2000 and 2002 (https://cls.ucl.ac.uk/cls‐studies/millennium‐cohort‐study/). For this study, we included data collected at the ages of 14 (2015, [SN 8156]) and 17 (2018, [SN 8682]), which can be accessed via the UK Data Service (https://ukdataservice.ac.uk/). The BHRCS (Salum et al., 2015) is an accelerated cohort study consisting of 2,511 young people (community = 958; high family risk for mental health problems = 1,553) from 57 schools in Brazil (22 in Porto Alegre and 35 in São Paulo). Participants were aged 6–14 years old at enrolment, with initial recruitment occurring between 2009 and 2010, and follow‐up assessments at 3 years (*n* = 2,010) and 8 years later (*n* = 1,905). Our analytic sample consisted of all individuals with valid data on our mental health outcome, and at least one of the social connection predictors.

### Measure harmonisation

To enable valid comparisons across the UK and Brazilian samples, equivalent variables were identified and retrospectively harmonised across the two studies. This process was undertaken by three authors (MH, BM and EM) over multiple rounds of screening and harmonisation. BM and EM were familiar with the MCS data, and MH was familiar with the BHRC data from previous work. The harmonisation process followed five steps:

1. First the authors BM and MH individually screened the MCS and BHRC metadata to identify and extract all variables in each cohort relating to social connection (referring to existing conceptual frameworks by Holt‐Lunstad, 2021 and Mansfield et al., 2023) and mental health. The metadata for these variables were transferred into an Excel sheet.
2. After the first round of screening and metadata extractions, the authors independently cross‐checked the metadata of the other country's cohort study to reduce selection biases and cross‐check all possible variables that had been identified in the first round of screening and extraction.
3. Once all relevant metadata were extracted, BM and MH conducted the first round of item matching based on perceived construct similarities. BM and MH are both mental health researchers with a clinical background. After the first round of item matching, to ensure that the identified variables were measuring the same constructs in both studies, all three authors had regular meetings to discuss the identified item matches and clarify any uncertainties regarding the matches until all authors agreed.
4. As a further check of face validity, we tested the semantic similarity of matched items using the Harmony online harmonisation tool (McElroy et al., 2024; Wood, Moltrecht, Hoffmann, Ploubidis, & McElroy, 2022). This tool uses the Sentence‐BERT (Reimers & Gurevych, 2019) model to calculate similarity scores between item pairs based on their semantic similarity. Harmony produces cosine similarity scores (Hcos) ranging from 0 to ±1, with values closer to 1 indicating semantically similar strings of text. For the Strength and Difficulties Questionnaire (SDQ) items, we achieved an average cosine similarity score of Hcos = 0.77, ranging from 0.61 to 0.92. For the ten social connection variables, we achieved an average cosine similarity score of Hcos = 0.59 ranging from 0.35 to 0.69 between the Brazil and the UK items, with one item pair, while correct having a notably lower match (‘current legal marital status’ and ‘a mãe biológica da criança está no momento’).
5. Once all possible and relevant items from both studies were extracted, all three authors discussed how to recode response options to ensure comparability for analyses. For example, in both cohorts, children were asked if they experienced being bullied. In the BHRCS, responses were reported as a simple yes/no. In the MCS, responses were indicated on frequency scale, ranging from 1 (most days) to 6 (never). Data in the MCS were therefore recoded to create a binary ever/never variable that was operationally similar to the equivalent variable in BHRCS.

An overview of the harmonised items and their rated similarity scores is provided in the Supporting Information.

### Exposure variables: social connection factors

We identified and selected the final list of social connection factors by reviewing the existing literature, involving lived experience experts and identifying and harmonising items relating to elements of social connection across the two studies (Table S1).

Based on the definition by Holt‐Lunstad et al. (2017), we included the 12 social connection factors across the following three social connection domains:

*Structural social connection*

- Household size (count)
- Number of siblings or half‐siblings in household
- Mother marital status (1 = married or living with biological parent; 2 = separated living with someone else; 3 = separated/divorced; 4 = single parent; 5 = widow and other)
- Self‐reported ‘having one good friend’ (yes/no)
- Death of parent or caregiver (no/yes)
- Moving address since last interview (no/yes)

*Functional social connection*

- Self‐reported bullying by others (never/ever)
- Self‐reported bullying of others (never/ever)

*Quality Social Connection*

- Parent‐reported ‘gets on with friends’ (no difficulties/difficulties)
- Main caregiver–child relationship (close/not close)

In MCS, the above variables were assessed at the age of 14, except for the parent‐reported assessment of ‘how well does child get on with their friends’, which was assessed at the age of 11. For BHRCS, these variables were taken from the second sweep (9–17 years; *M* = 13.5; *SD* = 1.93). A detailed list of variables and how they were recorded can be found in the Supporting Information.

### Outcome: adolescent mental health difficulties

Internalising and externalising difficulties were assessed with the self‐report version of the emotional and behavioural problems subscales (five items each) of the Strength and Difficulties Questionnaire (SDQ) in both studies (Goodman, Ford, Simmons, Gatward, & Meltzer, 2000). Higher scores suggest greater difficulties. The SDQ has been shown to have good validity and reliability scores in both countries. In MCS, the SDQ data were collected at the age of 17. For BHRCS, we used SDQ data for the ages 13–23 (*M* = 18.2; *SD* = 1.99).

### Covariates

We adjusted all models for the following covariates: parent‐reported adolescent SDQ score at prior sweep (SDQ), sex (male; female) ethnicity (majority vs. minority), maternal education (complete secondary education degree vs. less than secondary education degree),

^1^ location (urban vs. rural) and age (see the Supporting Information for more detail).

### Statistical analysis

Initial data analysis was conducted to provide sample characteristics and summary statistics of the exposure and outcome variables in both datasets. We tested the pooled sample for country‐level differences in sociodemographic characteristics and the exposure and outcome variables. We conducted a series of confirmatory factor analyses to test measurement invariance of the SDQ between both countries. Subsequently, we conducted separate regression analyses with one of the social connection factors at the age of 14 as our exposure and adolescent internalising and externalising difficulties at the age of 17 as the outcome. To correct for multiple testing, we adjusted *p*‐values using Benjamini–Hochberg method (Benjamini & Hochberg, 1995). We estimated a base model using covariates only to test how much each adds to predicting later internalising and externalising difficulties. All analyses were conducted in R version 4.3.2.

### Weights and missing data

Attrition weights were available for both cohorts and were applied to correct for missing data. MCS includes design weights that were applied to produce nationally representative estimates. For BHRCS, weighting variables to correct for the oversampling procedure (Martel et al., 2017) and attrition weights (using inverse probability weights) to account for follow‐up missingness were applied. Detailed information about BHRCS weights can be found on the OSF profile https://osf.io/ktz5h/.

To address sample size discrepancies between the studies, we calculated two additional weight variables. The first represents the ratio of the sample size in MCS to the sample size in BHRCS. We used this multiplied weight (oversampling*attrition*sample size) in the main pooled analysis. The second weight was computed using the same ratio but specifically for individuals residing in urban areas so the sample size in the BHRCS could be matched with urban MCS. This weight (oversampling*attrition*urban sample) was used for sensitivity analysis including only urban sample and for subgroup analysis (each study separated). For this analysis, we included only those observations for which a prior weight had been calculated.

### Sensitivity analysis

We estimated all models in each cohort dataset and in the pooled data. We also conducted unadjusted and unweighted regression models to understand at what level the main predictors were confounded by covariates, representativeness and use of weights. Due to the BHRC only containing participants living in urban areas, we conducted additional analyses restricting the MCS sample to participants from urban areas only.

## Results

### Sample characteristics

We included data from 11,756 participants with *N*_B_ = 2010 from Brazil and *N*_UK_ = 9,746 from the UK. When testing for sample differences between the two countries we found various significant differences for most sociodemographic characteristics, covariates, exposure variables and mental health outcomes (see Table 1). In comparison to BHRC, we found in MCS: more mothers had completed a secondary degree (61% vs. 46%), more participants from an ethnic majority group (88% vs. 52%), more mothers were married or living with the biological parent (60% vs. 49%), more parents reported that their child had difficulties keeping friends (34% vs. 6.8%), more children had been bullied by others (50% vs. 30%) or had bullied others (28% vs. 13%). In BHRC, more children had experienced the death of a parent (4.9% vs. 0.8% in MCS), had recently moved address (29% vs. 17% in MCS) and more parents reported that their child did not have at least one good friend (11% vs. 3.2% in MCS). Generally, adolescents in the BHRC had higher total scores on all SDQ subscales.

**Table 1:** Sociodemographic characteristics

Characteristic	BHRCS, *N* = 2,010		MCS, *N* = 9,746		*p*‐value
	Unweighted	Weighted	Unweighted	Weighted	
Biological sex at birth					.034
Male	1,125 (56%)	55.0%	4,671 (50%)	51.0%	
Female	885 (44%)	45.0%	4,738 (50%)	49.0%	
Missing	0	–	337	–	
Age in years	13.50 (1.92)	13.21 (1.89)	14.25 (0.34)	14.26 (0.35)	<.001*
Maternal education					<.001*
Less than secondary degree	1,135 (58%)	54.0%	3,484 (37%)	39.0%	
Completed secondary degree	826 (42%)	46.0%	5,874 (63%)	61.0%	
Missing	49	–	388	–	
Ethnicity					<.001*
Majority	1,107 (55%)	52.0%	7,905 (82%)	88.0%	
Minority	903 (45%)	48.0%	1,792 (18%)	12.0%	
Missing	0	–	49	–	
Location					<.001*
Rural	0 (0%)	0.0%	2,286 (24%)	24.0%	
Urban	2,010 (100%)	100.0%	7,441 (76%)	76.0%	
Missing	0	–	19	–	
SDQ internalising (P)	3.82 (2.74)	3.40 (2.59)	1.99 (2.11)	1.96 (2.11)	<.001*
Missing	1	–	1.477	1.274	
SDQ externalising (P)	2.39 (2.21)	2.16 (2.13)	1.33 (1.57)	1.35 (1.62)	<.001*
Missing	1	–	1.446	1.279	
SDQ total (P)	13 (8)	11 (7)	8 (6)	8 (6)	<.001*
Missing	1	–	1.309	1.185	
No. of people living in household	4.28 (1.44)	4.26 (1.42)	4.43 (1.31)	4.30 (1.23)	.024
Missing	5	7	0	0	
Siblings living in household	1,137 (57%)	55.0%	8,460 (87%)	85.0%	<.001*
Half‐siblings living in household	500 (25%)	23.0%	1,133 (12%)	13.0%	<.001*
Poor quality of maternal–child relationship					.002*
Extremely close	847 (42%)	45.0%	4,191 (47%)	47.0%	
Very close	898 (45%)	45.0%	3,658 (41%)	40.0%	
Somewhat close	237 (12%)	9.1%	1,104 (12%)	13.0%	
Not close	22 (1.1%)	0.7%	59 (0.7%)	0.6%	
Missing	6	–	734	–	
Maternal marital status					<.001*
Married/living with bio. parent	944 (48%)	49.0%	5,735 (62%)	60.0%	
Separated/living with someone else	352 (18%)	18.0%	705 (7.7%)	8.1%	
Single parent	318 (16%)	16.0%	1,327 (14%)	16.0%	
Separated/divorced	284 (14%)	14.0%	1,337 (15%)	15.0%	
Widow	66 (3.4%)	2.7%	96 (1.0%)	0.9%	
Missing	46		546	–	
Paternal death					<.001*
Not dead	1,906 (95%)	95.0%	9,658 (99%)	99.0%	
Dead	104 (5.2%)	4.9%	88 (0.9%)	0.8%	
Difficulty getting along or keeping friends (P)					<.001*
No difficulty	1,810 (90%)	93.0%	6,180 (68%)	66.0%	
Difficult	194 (9.7%)	6.8%	2,961 (32%)	34.0%	
Missing	6	–	605	–	
Child ever bullied	247 (14%)	13.0%	2,655 (28%)	28.0%	<.001*
Missing	216	–	317	–	
Child ever been bullied	582 (32%)	30.0%	4,651 (49%)	50.0%	<.001*
Missing	211		322	–	
Child does not have at least one good friend^r^ (P)	265 (13%)	11.0%	263 (3.1%)	3.2%	<.001*
Missing	0	–	1.290	–	
I do not have at least one good friend	112 (6.2%)	6.0%	287 (3.0%)	3.3%	<.001*
Missing	215	–	198	–	
Moved to different house/city	581 (29%)	29.0%	1,271 (14%)	17.0%	<.001*
Missing	0	–	550	–	

Unweighted: *n* (%) or mean (*SD*); weighted: % or mean (*SD*). (P) = parent reported, ^r^reverse coded.

Chi‐squared test with Rao & Scott's second‐order correction; Wilcoxon rank‐sum test for complex survey samples; tests performed on weighted values.

* Significant.

### Measurement invariance testing and scoring

Our confirmatory factor analysis of the SDQ scale suggested good model fit in both countries (Table S2). Increased restrictions invariance testing in the pooled dataset suggested that there was acceptable measurement invariance between the country on a configural, metric and scalar level for parent (Table S3) and self‐reports (Table S4). We included factor scores of scalar level models for the subsequent analyses. SDQ latent variable means for BHRCS are set to zero by default. For parent reports at baseline and self‐reports at follow‐up, lower latent means for internalising (ɳ = −.416 and ɳ = −.158, respectively) and externalising symptoms (ɳ = −.306 and ɳ = −1.021, respectively) were observed when comparing MCS with the BHRCS.

### Pooled regression analyses: independent associations of social connection and mental health difficulties

**Figure 1:**
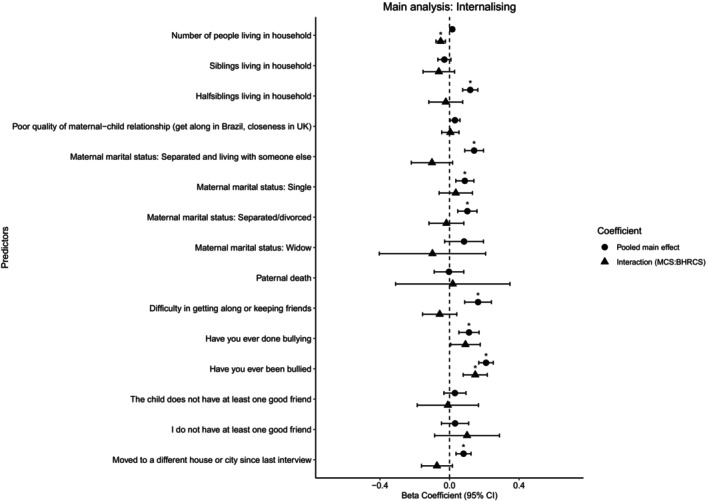
Main and interaction effects of social connection factors on internalising symptoms based on adjusted regression models

Regression analyses in the pooled dataset indicated that six of the 12 social connection factors were associated with greater internalising difficulties at a later stage (see Figure 1 and Tables S5–S16). This included three structural (‘living with half‐siblings’ β = 0.119; 95% CI [0.076, 0.163], ‘moving house or city’ β = 0.081; 95% CI [0.037, 0.124], and different mother *marital statuses*: ‘separated living with someone else’ β = 0.141; 95% CI [0.088, 0.195], ‘single’ β = 0.088; 95% CI [0.036, 0.14] and ‘divorced’ β = 0.103; 95% CI [0.047, 0.158]), two functional (*bullying others* β = 0.112; 95% CI [0.054, 0.170] and *being bullied* β = 0.21; 95% CI [0.169, 0.252]) and one quality social connection factor (*difficulties keeping friends* β = 0.164; 95% CI [0.087, 0.241]).

**Figure 2:**
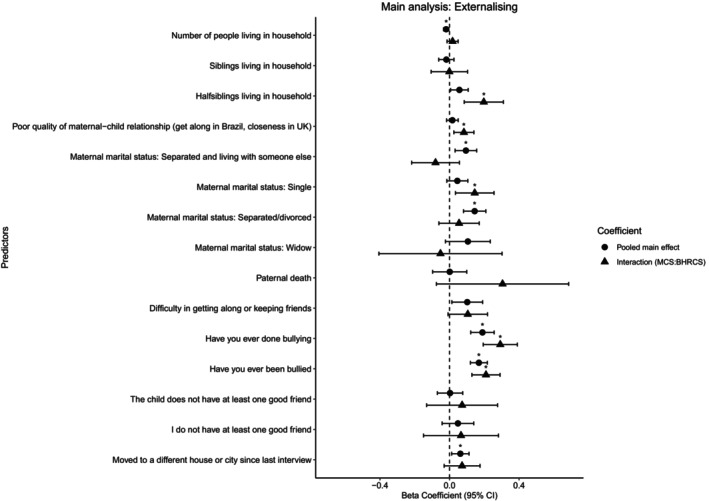
Main and interaction effects of social connection factors on externalising symptoms, based on adjusted regression models

For externalising symptoms (see Figure 2 and Tables S5–S16), we found similar associations for three structural (‘number of people living in household’ β = −0.019; 95% CI [−0.035, –0.004], ‘moving house or city’ β = 0.062; 95% CI [0.012, 0.112] and mother's *marital status*: separated and living with someone else β = 0.094; 95% CI [0.032, 0.156] and separated/divorced β = 0.145; 95% CI [0.080, 0.209]) and two functional (‘bullying others’ β = 0.189; 95% CI [0.122, 0.257] and ‘being bullied’ β = 0.169; 95% CI [0.120, 0.217]) factors. While the majority of the social connection factors seemed to relate similarly to the two mental health outcomes, we also found some small differences for ‘number of people in households’ (i.e. externalising only), ‘number of half‐siblings in household’ (internalising only) and ‘difficulty keeping friends’ (i.e. internalising only).

### Interactions: cross‐country comparisons

We also found significant country‐level interaction effects, where two social connection factors were associated with greater internalising symptoms in one country but not the other (see Figure 1). For Brazil, ‘number of people in household’, a structural factor, had a greater association with internalising problems β = −0.051; 95% CI [0.003, 0.029] than in MCS. In the UK, however, we found that ‘being bullied’ β = 0.148; 95% CI [0.079, 0.217], a functional factor had greater associations with internalising problems. For externalising symptoms (see Figure 2), we found country‐specific associations for five factors from all three domains. Two structural (‘half‐siblings living in household’ β = 0.197; 95% CI [0.084, 0.310] and ‘maternal *marital status* of being *single*’ β = 0.145; 95% CI [0.034, 0.256]), two functional (‘bullying others’ β = 0.292; 95% CI [0.194, 0.390] and ‘being bullied’ β = 0.21; 95% CI [0.129, 0.291]) and one quality factor (‘quality of mother–child relationship’ β = 0.083; 95% CI [0.025, 0.140]) had stronger associations with externalising symptoms in UK adolescents. Complete adjusted and weighted results can be found in Tables S5–S16 for internalising and externalising symptoms.

### Sensitivity analyses

We conducted three different sensitivity analyses, including (a) separate regression analyses for each country's dataset, (b) regression analyses in a pooled dataset where MCS only contained participants living in urban areas and (c) regression analyses without covariates and adjustments with weights. For all sensitivity analyses, we found similar patterns as for the association between social connection factors and subsequent internalising and externalising symptoms in adolescents (Figures S1 and S2, respectively). More specifically, we found the same independent association for ‘half‐siblings living in household’, ‘difficulties keeping friends’, ‘bullying others’, ‘being bullied’ and ‘separated’ or ‘divorced’ marital status on internalising symptoms across all main and sensitivity analyses. For externalising symptoms, we found that ‘bullying others’, ‘being bullied’ and ‘divorced’ marital status were significant predictors across all sensitivity analyses. The functional factor ‘being bullied’ was most consistently related to higher internalising and externalising problems in both countries, but with a greater impact on MCS adolescents. Detailed findings from the sensitivity analyses are presented in the Supporting Information see Figure S1 (internalising) and S2 (externalising) and Tables S17 to S28 for BHRCS, Tables S29 to S40 for MCS, Tables S41 to S52 for unadjusted analysis, and Tables S53 to S64 for analysis in urban population only.

## Discussion

We investigated whether factors of different social connection domains (i.e. structural, functional and quality) experienced during early adolescence were associated with later internalising and externalising problems and if these associations vary across two countries UK and Brazil. Retrospective measurement harmonisation allowed us to pool data from two large cohort studies conducted in both countries. Our regression models in the pooled data showed that structural social connection factors relating to non‐intact family structures (e.g. living with half‐sibling, mother being separated, divorced or single) and functional factors, especially *being bullied* were consistently associated with greater adolescent mental health difficulties in both countries.

Our findings suggest an association of bullying with later mental ill‐health which is in line with the vast amount of research that has proven the detrimental and long‐term effects of bullying (for both being bullied and bullying others) on mental ill‐health (Arseneault, 2018; Ganesan et al., 2021; Klomek, Sourander, & Elonheimo, 2015). We also found specific country‐level differences for being bullied and bullying others which was more frequently reported by UK adolescents (49% vs. 32%) and had a greater association with later internalising symptoms in UK adolescents but not in Brazil. Not many studies have investigated cross‐country differences of bullying or other social connections factors and mental health outcomes. A cross‐national examination among young adults in seven countries demonstrates that differences in types of bullying might explain the results. In England, the type of bullying that is practised is higher in ‘left out of activities’, which is related to social exclusion. On the other hand, in Argentina, for example, ‘made fun of how my body or face looks’ is a more frequent type of bullying (Labella et al., 2024). Even though we have not measured type of bullying and the mentioned study did not involve Brazil, different types of bullying (i.e. being left out of activities rather than teased by how someone looks) might explain why bullying in the UK predicts higher levels of internalising problems than in Brazil. Nonetheless, it is important to highlight that practising or being bullied were associated with internalising and externalising problems in both countries and the interactions show additional context‐dependent effects. We expect that pathways between risk factors, such as bullying behaviour, and mental health outcomes differ between countries and recommend that future research uses a combination of qualitative (Wahid et al., 2022) and quantitative longitudinal studies (Ganesan et al., 2021) to shed light on these differences, so that context‐sensitive interventions can be implemented.

In terms of non‐intact family structures (e.g. parent separation/divorce, living with half‐siblings), which tend to coincide with family conflict and greater levels of instability, the negative impact on child and adolescent mental health outcomes has repeatedly been shown (Eriksson, Cater, Andershed, & Andershed, 2011; Goodman, Fleitlich‐Bilyk, Patel, & Goodman, 2007; Lee & McLanahan, 2015; McCulloch, Wiggins, Joshi, & Sachdev, 2000). We found specific country‐level associations for the two structural factors ‘single mother’ and ‘living with a half‐sibling’ with externalising symptoms in MCS. MCS adolescents seemed to be more strongly affected by non‐intact family structures than Brazilian adolescents. One possibility could be that this finding is a statistical artefact due to differences in sample sizes, with MCS having a much larger sample (*N*_UK_ = 9,746 and *N*_B_ = 2,010); however, we tested for this in our sensitivity analyses, which provided similar findings. Another possible explanation, as research from the UK has shown, is that parental divorce/separation (but not parental death) had negative consequences for young people's socioemotional development but that these effects were less pronounced in non‐white children compared to white children (Brand, Moore, Song, & Xie, 2019; Lee & McLanahan, 2015; Rodgers, Power, & Hope, 1997). This finding has been attributed to non‐white families (a minority group in the UK) experiencing greater socioeconomic disadvantages compared to white families, and therefore changes in socioeconomic positions due to parental separation are attenuated in non‐white‐ethnic groups (Lee & McLanahan, 2015). As we adjusted our analysis for skin colour/ethnicity, another explanation could be that other cultural differences explain these associations. For instance, some ethnic groups, such as Latinos and Black Africans, seem to rely more on extended family networks for support and are therefore better equipped to cope with instability in the nuclear family structure. Furthermore, the type of support provided seems to differ in that they seem to provide more practical support (e.g. childcare, housework), whereas some white‐ethnic families (in UK context) tend to provide more emotional support. Although this past research aligns with our findings, previous studies have mostly compared ‘white’ with ‘non‐white’ groups living in the same – often high income – country and little attention has been given to cultural facets of ethnicity (Lee & McLanahan, 2015). We are not aware of any study that has specifically researched country‐level differences in family factors and mental health outcomes. To this day, there are considerable limitations regarding studies exploring cross‐country variations of risk pathways even though we can expect these to differ across countries. On a different but related note, we want to mention that in BHRC individuals were asked about their skin colour, whereas in MCS participants were asked about their ethnicity. While these seem like similar concepts, they are also slightly different, which may have contributed to bias and may limit the comparability of the data (e.g. in Brazil someone can have ‘white skin colour’ but identify as Latino).

In relation to cross‐country comparisons, we also found fewer overall associations between our included social connection factors and mental health outcomes in the Brazilian sample in comparison to the UK study. This might be due to differences in sample sizes, although we accounted for this by running models with and without adjustment weights. Furthermore, as is the case for subjective, self‐report measures there is a possibility that these are interpreted differently and residual differences in how the concepts are measured exist. It could also be related to residual confounding specifically for each cohort. Due to the scarcity of research pooling data from different countries, there is limited evidence and guidance available for the recommended choice of data analytical approaches, and how to examine and address country‐level differences. We conducted multiple analyses, with and without weights and adjustments to get a comprehensive understanding of the data. Thus, as a limitation and despite our efforts to minimise sampling bias by using scalar‐invariant SDQ scores, sampling and attrition weights and replication analysis, we cannot rule out that unmeasured sampling differences may account for some of the associations found in the present study. Such biases are inherent in individual‐level data integration, and global surveys using the same protocols could minimise such problems.

Lastly, our harmonisation approach combined with the LEE consultations conducted in both countries helped us identify relevant variables for our research question and also highlighted where the two datasets have constraints. Our list of social connection factors is not comprehensive and some variables were not included if they were only available in one study. Additionally, most of our data captured structural social connection factors and insights from functional and quality factors were limited. As mentioned before, our LEE experts particularly emphasised the lack of data around the qualitative experiences of social connections as a limitation. We suggest that future research looks at multiple factors across all social connection domains. We are unaware of existing data from longitudinal population surveys that provide this information, so there might be a need to collect these data in a new study.

## Conclusion

We found that social connection factors, we primarily investigated factors from the structural domain, contributed to adolescent internalising and externalising difficulties in both countries. Some factors relating to bullying and family composition seem to play a unique role in UK adolescents. Limited data were available for functional and quality social connection factors; hence, our evidence is limited around these. Cross‐country comparison studies are overall scarce; however, we were able to add to the existing evidence base by harmonising and pooling data from two large, longitudinal cohort studies from these two countries. There is still limited understanding of how risk pathways compare across countries, and future research needs to uncover cross‐regional differences to meaningfully inform global mental health efforts.

## Ethical approval

This study used secondary data from the Millennium Cohort Study and the Brazilian High Risk Cohort Study. Millennium Cohort Study has gained ethical approval for all data collection points. For data used in this study, ethical approval was given by the National Health Service (NHS) Research Ethics Committee (REC) for age 11 and 14 data collection (registration numbers [11/YH/0203] and [13/LO/1786]). Ethical approval for age 17 was obtained from the National Research Ethics Service (NRES) Research Ethics Committee (REC) North East – York (REC ref: 17/NE/0341). Written consent was obtained from parents and assent from children when participants were younger than 16 years old. As of age 16 participants provided their own verbal consent and parental consent was not required. Irrespective of any consent or assent, individuals were able to refuse to participate in any element of a survey or withdraw from the study at any time by simply expressing the wish to do so. The Brazilian High Risk Cohort Study has gained ethical approval by the ethics committee of the University of São Paulo [IORG0004884/National Council of Health Registry number (CONEP): 15.457/Project IRB registration number: 1132/08]. Written consent was obtained from parents of the participants as well as from those participants who were able to read, write and understand the written consent. From others, verbal agreement was obtained.

**Key points**

- Adolescent mental health problems are highly prevalent across the globe. Most research has been conducted in high‐income countries, and differences to low‐ and middle‐income countries are often unknown.
- Retrospective measurement harmonisation and pooling of data from different countries can help uncover how important risk pathways differ across countries to subsequently inform mental health interventions.
- Social connection factors are associated with adolescent mental health difficulties in both Brazil and the United Kingdom but country‐specific associations exist which need to be researched further.

## Supporting information


**Figure S1.** Sensitivity analysis for internalising symptoms.
**Figure S2**. Sensitivity analysis for externalising symptoms.
**Figure S3**. Jam board completed by young advisors.
**Figure S4**. Jam board completed by young advisors.
**Table S1**. Overview of harmonised items.
**Table S2**. Confirmatory factor analysis scalar‐invariant model of SDQ domains across different informants.
**Table S3**. Measurement invariance of parent‐report SDQ across study type.
**Table S4**. Measurement invariance of self‐report SDQ across study type.
**Tables S5–S16**. Pooled regression analysis: *respective social connection factor*.
**Tables S17–S28**. BHRCS regression analysis: *respective social connection factor*.
**Tables S29–S40**. MCS regression analysis: *respective social connection factor*.
**Tables S41–S52**. Unadjusted regression analysis: *respective social connection factor*.
**Tables S53–S64**. Urban population regression analysis: *respective social connection factor*.
**Table S65**. Overview of *p*‐values pre‐ and post‐adjustments.

## Data Availability

The MCS data that support the findings of this study are openly available via the UK DataService (https://ukdataservice.ac.uk/) at 10.5255/UKDA‐SN‐8156‐7 and http://doi.org/10.5255/UKDA‐SN‐8682‐2 and under the following reference numbers (age 14 [SN 8156]; age 17 [SN 8682]). BHRCS individual‐level data are available upon request to the BHRCS research committee, by following the instructions and filling the research form available at https://osf.io/ktz5h/wiki/home/. Data dictionary is available at https://osf.io/ktz5h/wiki/DataDictionaries/ and https://osf.io/w3jr4 to direct download.

## Endnote

1 With reference to the International Standard Classification of Education (ISCED), we considered everything below ISCED level 4 as ‘less than secondary education degree’ and above ISCED level 4 as ‘completed secondary education degree’.
