# Supplementary material for: Social connection and its prospective association with adolescent internalising and externalising symptoms: an exploratory cross‐country study using retrospective harmonisation
Source: J Child Psychol Psychiatry. 2024 Dec 7;66(5):725–36. doi: 10.1111/jcpp.14080 (PMC12018293; doi:10.1111/jcpp.14080)

# Supporting Information

Table of Contents

[Supporting Information 1](#_Toc180677528)

[Sensitivity analyses 2](#_Toc180677529)

[Sensitivity analyses results 3](#_Toc180677530)

[Measurement Harmonisation 5](#_Toc180677531)

[Measurement Invariance Testing 10](#_Toc180677532)

[P-Values pre and post adjustments 56](#_Toc180677533)

[Lived Experience Workshop 61](#_Toc180677534)

[**Examples Part 1 activities:** 61](#_Toc180677535)

[**Examples Part 2 activities** 61](#_Toc180677536)

## Sensitivity analyses

We conducted three different sensitivity analyses, including a) separate regression analyses for each country’s dataset, b) regression analyses in a pooled dataset where MCS only contained participants living in urban areas and c) regression analyses without covariates and adjustments with weights.

***Single country analyses.*** Overall, we found fewer associations between the assessed social connection factors and adolescent mental health outcomes in the Brazil study than in the UK sample. Regression models conducted in the BHRC data showed that only one of the social connection factors, ‘*having been bullied’* was associated with later internalising problems (Figure S1 and tables S17 to S28). Additionally, we found that ‘*being bullied’*, and ‘*bullying others’* were associated with later externalising problems, but none of the other social connection factors (Figure S2 and tables S17 to S28). Regression analysis in the MCS cohort data suggested that all but four social connection factors were associated with later internalising difficulties (Figure S1 and tables S29 to S40). We found that ‘*number of people in household*’ and ‘*siblings living in household’* were associated with decreased internalising symptoms, while ‘halfsiblings in household’ was linked to higher internalising symptoms. Additionally, ‘*poor mother-child relationship’*, mothers being ‘*single’* or ‘*divorced’* from parent, ‘*child having difficulty keeping friends’*, ‘*bullying others’* and ‘*being bullied’* were all associated with higher internalising problems later on. ‘*Paternal death*’, ‘*moving address’* and parent- and self-reported indication of the ‘*child not having at least one good friend*’ were not associated with subsequent internalising problems. The same social connection factors, and ‘*having moved address’* were linked to an increase in externalising problems (Figure S2 and tables S29 to S40). However, we also found that some of associations, primarily in the internalising regression models, became insignificant, but keeping their effect trends, after adjusting the p-values (Table S65).

***Unadjusted regression analyses.*** The unadjusted regression models (without weights and covariates) showed similar patterns in terms of the associations between the social connection factors and subsequent internalising problems (Figure S1 and tables S41 to S52). However with two additional factors being significant (‘poor mother-child relationship’ and parent reported ‘child does not have at least one good friend’). The same two factors became significant in the unadjusted regression model for externalising symptoms (Figure S2 and tables S41 to S52). Additionally, the unadjusted pooled regression models suggested two more associations of ‘living with half-siblings’ and ‘difficulties keeping friends’ with later externalising problems. In summary, the unadjusted regression models showed similar patterns of associations, but with 2 to 4 additional social connection factors having significant associations with subsequent mental health outcomes. In terms of country-specific effects, we found similar association patterns as in the adjusted regression models, with a few social connection factors now showing greater or significant associations: ‘number of siblings living in household’ and ‘difficulties keeping friends’ were more strongly associated with later internalising symptoms in Brazil adolescents, but not UK adolescents.

***Regression analysis with urban only sample***. Regression analysis in the pooled dataset containing only MCS participants from urban areas suggested the same interaction effects and similar main effects to be associated with later internalising symptoms (Figure S1 and tables S53 to S64). For externalising symptoms there were fewer main and country-level effects in the urban only sample, only indicating associations with ‘bullying others’ and ‘being bullied’ (Figure S2 and tables S53 to S64).

## Sensitivity analyses results

We conducted three sensitivity analyses of which the results are shown below. Detailed findings can be found in the online excel files: Regression analyses for each cohort (MCS and BHRCS) separately, unadjusted regression analyses, and only [urban](https://docs.google.com/spreadsheets/d/16jMFy0FzdDtkcDqlaDieanRDzRhd01UH/edit?rtpof=true#gid=1998562777) participants.


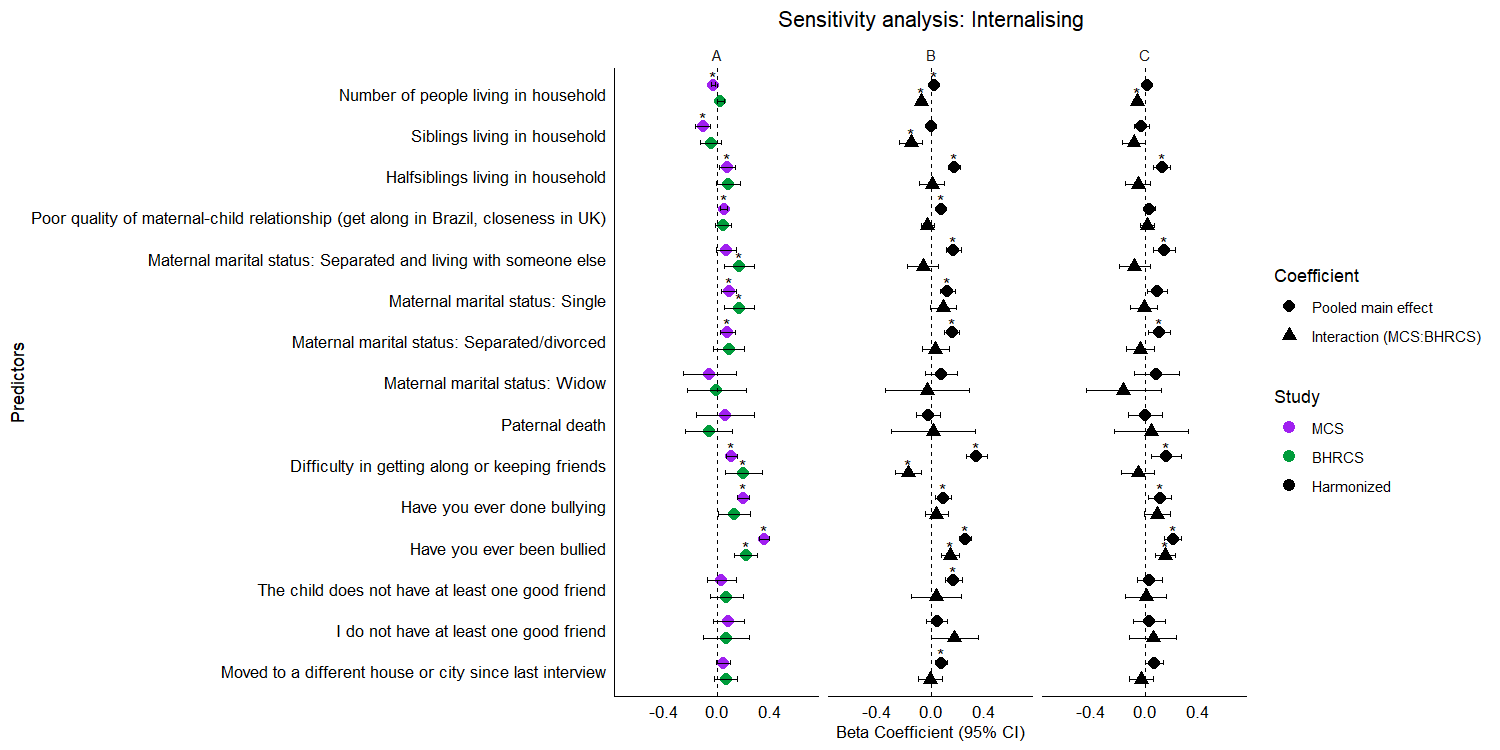


*Figure S1 – Sensitivity analysis for internalising symptoms. A. Regression analysis by study; B. Unadjusted regression analysis; C. Urban regression analysis.*


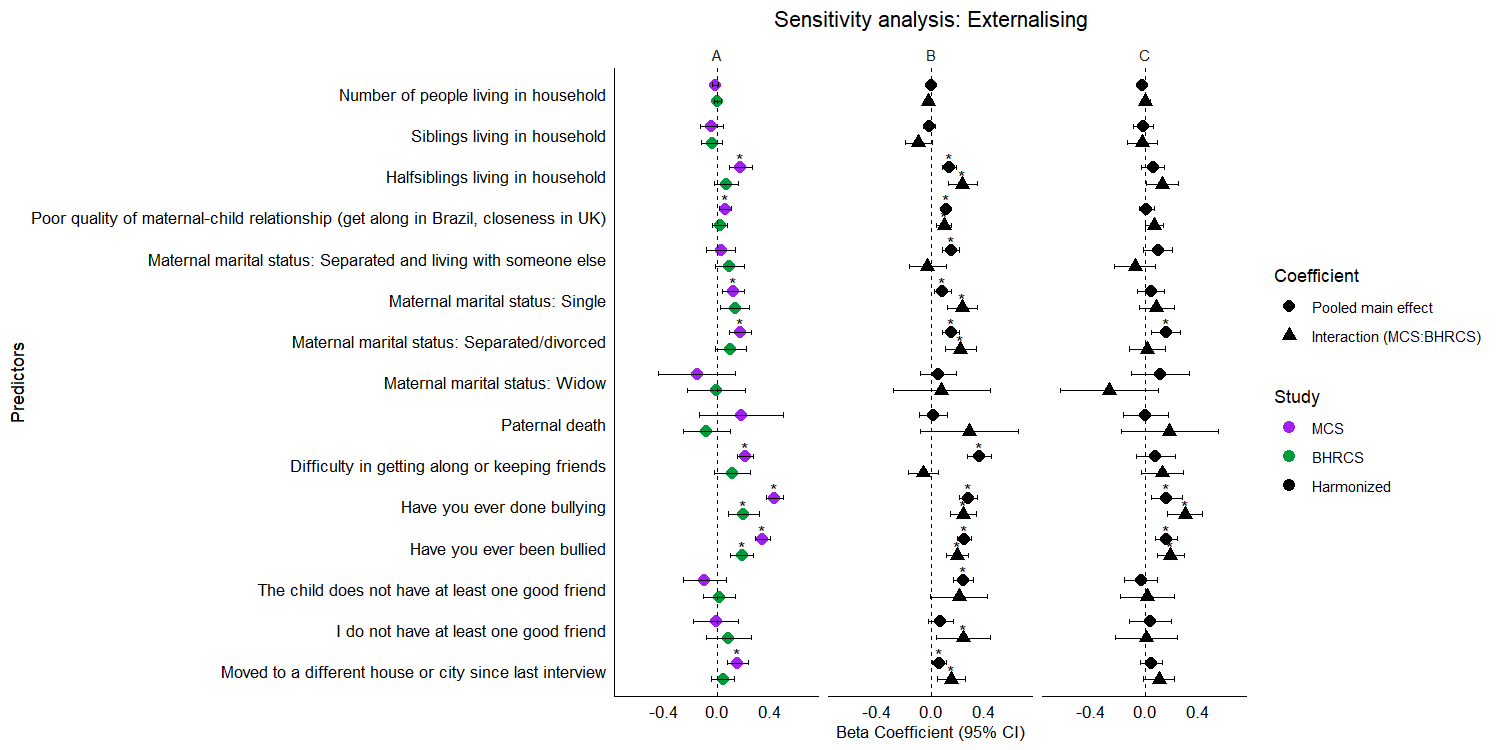


*Figure S2 - Sensitivity analysis for externalising symptoms. A. Regression analysis by study; B. Unadjusted regression analysis; C. Urban regression analysis.*

## Measurement Harmonisation

Manual measurement harmonisation was conducted by three authors (MH, BM, EM) over multiple rounds of screening, extraction and harmonisation. BM and EM were familiar with the MCS data and MH was familiar with the BHRC data from previous work. We followed the steps as described in the manuscript. Here we present Table S1 which shows how each variable was matched across the two cohorts, how the response options were recoded and which similarity score (*Hcos*) we received when using the online Harmony tool.

TABLE S1. Overview of harmonised items

| **BHRCS** | | | | | | |  | **Pooled dataset** |  | | **MCS** | | |  | **H Cosine** |
| --- | --- | --- | --- | --- | --- | --- | --- | --- | --- | --- | --- | --- | --- | --- | --- |
| **Question** | | **Question in Portuguese** | | **Response option** | | **Recoding** |  | **Variable name & final coding** |  | | **Recoding** | **Response option** | **Question in English** |  |  |
| **Concept: Household size** | | | | |  |  |  |  | |  |  |  |  |  |  |
| How many people live in the house where the child lives? | | Quantas pessoas moram na casa onde o jovem mora? | | numerical | | n/a |  | *peoplehouse*  [numerical: 0-10+] |  | | n/a | 0- 15 | Number of people present in Household including Child |  | 0.634 |
| Number of biological siblings living with the child | |  | | numeric, computed from a checklist | | Brazil  0=0  >0=1 |  | *siblings*  [no=0, yes=1] |  | | UK  0=0  >0=1 | 0-11 | Number of sibs in HH plus CMs |  |  |
| Number of half siblings living with the child | |  | | numeric,computed from a checklist | | Brazil 0=0  Brazil >0 =1 |  | *halfsiblings*  [no=0, yes=1 ] |  | | UK 1=1  UK 2=0 | 1=At least 1 half sib in HH;  2=No half sibs in HH | Half siblings of CM in HH |  |  |
| **Concept: Mother child relationship** | | | | |  |  |  |  |  | |  |  |  |  |  |
| How does/did the child and the biological mother get along when they are/were together? | | Como a criança e mãe biológica se dão ou se davam quando estão/estavam juntas? | | 1= Very well, 2= Well, 3= More or less, 4= bad, 5= Very bad, 88= Does not apply, 99= Does not know | | Brazil 4\|5 = 3 Brazil 3= 2 Brazil 2= 1 Brazil 1= 0 |  | *Mcrelation*  3= not close,  2=somewhat close,  1= very close,  0= extremely close |  | | UK 4 = 0  UK 3= 1  UK 2= 2  UK 1= 3 | 1= Not very close  2= Fairly close  3= Very close  4 =Extremely close  5 = Don’t know /Don’t wish to answer | Overall, how close would you say you are to CM? |  | 0.613 |
| **Concept: Marital status** | | | | |  |  |  |  |  | |  |  |  |  |  |
| What is the marital status of the biological mother? | | A mãe biológica da criança está no momento: casada ou morando junto com o pai biológico, casada ou morando junto com companheiro, solteira, separada ou viúva? | | 1= Married or living with biological father, 2= Married or living with another partner,  3= Single,  4=Separated/Divorced, 5=Widow | | Brazil 1= 1  Brazil 2= 2  Brazil 3\|4= 3  Brazil 5= 4 |  | *Maritalstatus*  1= Married or living with biological parent 2= Separated and living with someone else 3= Separated /Divorced 4 =Single 5= Widow |  | | UK 1/6/=1  UK 2=2  UK 3=4  UK -1/4=3  UK 5=5 | -1= Legally separated,  1= Married,1st and only marriage,  2=Remarried, 2nd or later marriage  3=Single, never married, 4= Divorced, 5= Widowed, 6= A Civil Partn | Current legal marital status |  | 0.352 |
| **Concept: Parental death** | | | | |  |  |  |  |  | |  |  |  |  |  |
| Is the child's biological father known? | | O pai biológico da criança é desconhecido, conhecido ou falecido? | | 1= Unknown, 2= Known, 3= Deceased | | Brazil 3 = 1  Brazil 1\|2 = 0 |  | *Fatherstatus*  0= not dead 1= dead |  | | UK 3=1 "dead"  all other =0 | 1= Resident full-time in house, 2= Resident part-time in house, 3=Deceased, 4= Non-resident, in contact, 5=Non-resident, not in contact, 6=Non resident, contact not known | Natural father status |  | 0.685 |
| Is the child's biological mother known? | | A mãe biológica da criança é desconhecido, conhecido ou falecido? | | 1=Unknown, 2= Deceased, 3= Known | | Brazil 2 = 1, Brazil 1\|3 = 0 |  | *motherstatus*  0=not dead 1= dead |  | | UK 3=1 "dead"  all other =0 | 1=Resident full-time in house, 2= Resident part-time in house, 3= Deceased, 4=Non-resident, in contact, 5=Non-resident, not in contact, 6= Non resident, contact not known | Natural mother status |  | 0.693 |
| **Concept: Getting on with friends** | | | |  | |  |  |  |  | |  |  |  |  |  |
| Difficulty keeping friends | | Como é a criança para manter os amigos que fez? | | 0= Finds it harder than average, 1= average, 2= Finds it easier than average | | Brazil 0=1  Brazil 1\|2= 2 |  | *friendrelation*  0= No difficulty 1= Difficult |  | | UK 1\|2\|3= 1  UK 4\|5= 2 | 1=Most days, 2=at least once a week, 3= At least once a month,  4= Less often than once a month, 5= Never, 6= Don’t have any friends | How often do you argue or fall out with your friends? |  | 0.440 |
| **Concept: Bullying** | |  | |  | |  |  |  |  | |  |  |  |  |  |
| Have you ever **done** bullying? | | Você sabe se o seu(sua) filho(a) fez “bullying” alguma vez na vida? | | 0=No,1=Yes | | Brazil 0=0  Brazil 1=1 |  | *donebullying*  Yes=1 No=0 |  | | UK 1\|5=1  UK 6=0 | 1=Most days, 2=About once a week, 3= About once a month, 4=Every few months, 5=Less often, 6=Never | CM Often fights with other children or bullies them |  | 0.603 |
| If yes, and **this year**, did you do bullying? | |  | | 0=No,1=Yes | | Brazil 0=0  Brazil 1=1 |  | Yes=1 No=0 |  | | UK 1\|5=1  UK 6=0 | 1=Most days, 2=About once a week, 3= About once a month, 4=Every few months, 5=Less often, 6=Never | How often do you hurt or pick on other children on purpose? |  |  |
|  | |  | |  | |  |  |  |  | |  |  |  |  |  |
| Has the child ever been bullied? | | Você sabe se o seu(sua) filho(a) sofreu “bullying” alguma vez na vida? | | 0=No,1=Yes | | UK 1\|5=1  UK 6=0 |  | *beenbullied*  Yes=1 No=0 |  | | UK 1\|5=1  UK 6=0 | 1=Most days, 2=About once a week, 3= About once a month, 4=Every few months, 5=Less often, 6=Never | How often do other children hurt you or pick on you on purpose? |  | 0.671 |
| **Concept: One good friend** | | | | |  |  |  |  |  | |  |  |  |  | |
| Has good friend | | Tem pelo menos um bom amigo ou uma boa amiga | | 0= True  1= More or less true  2= False | | Brazil 2 = 1 UK "False" = 1  Brazil 1 = 2 UK "More or less true" = 1  Brazil 0 = 3 UK "True" = 0 |  | *pgoodfriend*  0 = Have a good friend, 1 = do not have a good friend |  | | Brazil 2 = 1 UK "False" = 1  Brazil 1 = 2 UK "More or less true" = 1  Brazil 0 = 3 UK "True" = 0 | 1 Not true 2 Somewhat true 3 Certainly true 4 Dont know/Dont wish to answer | CM has at least one good friend |  | 0.680 |
| Has good friend | | Tenho pelo menos um bom amigo ou uma boa amiga | | 0= True, 1= More or less true , 2= False | | Brazil 2 = 1 UK "False" = 1  Brazil 1 = 2 UK "More/ less true" = 1  Brazil 0 = 3 UK "True" = 0 |  | *sgoodfriend*  0 = Have a good friend, 1 = do not have a good friend |  | | Brazil 2 = 1 UK "False" = 1  Brazil 1 = 2 UK "More or less true" = 1  Brazil 0 = 3 UK "True" = 0 | 1 Not true 2 Somewhat true 3 Certainly true 4 Dont know/Dont wish to answer | I have one good friend or more |  |  |
| **Concept: Moving house** | | | |  | |  |  |  |  | |  |  |  |  |  |
| Since last interview, Changed/moved city ? | | Desde a última entrevista, mudou de cidade? | | 0 Não; 1 Sim | | moved |  | *moved*  1= Yes  0= No |  | |  | Yes/No | (6,7) Same address as last interview? |  | 0.555 |
| **Control variables** | |  | |  | |  |  |  |  | |  |  |  |  |  |
| **Concept: ethnicity** | |  | |  | |  |  |  |  | |  |  |  |  |  |
| Ethnicity | | Cor da pele da criança | | 1= White, 2= Black , 3= Between white and black (brown), 4= Indigenous, 5= Asian | | Brazil 1=0  Brazil 2\|5=1 |  | *ethnicity*  majority (0)  minority (1) |  | | UK0=1  UK 2\|6=1 | 1= White  2= Mixed  3= Indian  4= Pakistani and Bangladeshi  5= Black or Black British  6= Other ethnic group (inc. Chinese, other Asian) | Main respondents ethnic group: |  | n/a |
| **Concept: Child sex** | |  | |  | |  |  |  |  | |  |  |  |  |  |
| Child gender | |  | | 1=male, 2=female | |  |  | *sex*  1= male  2= female |  | | 1= male  2= female | 1 male  2 female | Sex |  | n/a |
| **Concept: Maternal education** | | | |  | |  |  |  |  | |  |  |  |  |  |
| Maternal education* | Qual o grau de instrução da mãe biológica no momento? | | 1= Without study, 2=Elementary school incomplete, 3= Elementary school completed, 4=Incomplete high school, 5= High school, 6= Incomplete higher education, 7= Higher education, 8= Higher education with postgraduate studies | | | Brazil 1\|4=1  Brazil 5\|8=2 |  | *Matedu*  1: less than complete secondary degree  2: complete secondary degree |  | | UK 1\|3=1  UK 4\|5=2 | 1 NVQ level 1  2 NVQ level 2  3 NVQ level 3  4 NVQ level 4  5 NVQ level 5  Overseas qual only | which, if any, of the vocational qualifications? |  |  |
| **Concept: Location** | | | |  | |  |  |  |  | |  |  |  |  |  |
| n/a | | n/a | | n/a | | all urban |  | *Location*  1 = urban  2= rural |  | | Rural/urban | Rural/urban | based on address at interview |  | n/a |

*Education pathways differ in both countries; we have differentiated between mothers with or without a secondary education degree. For the UK this means that based on the national vocation qualification (NVQ) levels every participant between NVQ levels 1 to 3 (up to A-levels) was considered as “less than secondary degree” and participants with NVQ levels higher than 3 (e.g. higher education certificate, higher apprenticeship) were categorized as “complete secondary degree”. With respect to the International Standard Classification of Education (ISCED) we considered everything below ISCED level 4 as “less than secondary education degree” and above ISCED level 4 as “completed secondary education degree

## Measurement Invariance Testing

We conducted confirmatory factor analyses for the SDQ items and tested for measurement invariance between the two cohort and for the parent and self-report data. A scalar-invariant correlated four-factor model was chosen. In the parent-report model, the correlation between internalising and externalising factors was 0.550 for the BHRCS group and 0.523 for the MCS group. In contrast, for the self-report model, the correlations between internalising and externalising were 0.691 and 0.358 for BHRCS and MCS respectively. Table S2 shows the factor loadings for each item in the pooled dataset and the scalar-invariant model fit indices.

**Table S2 - Confirmatory factor analysis scalar-invariant model of SDQ domains across different informants**

| **Variable** |  |  | **Factor loadings** | | | |
| --- | --- | --- | --- | --- | --- | --- |
|  |  |  | Parent-report | | Self-report | |
| Item content | Item number |  | Int | Ext | Int | Ext |
| Often complains of headaches, stomach-aches or sickness | 3 |  | 0.558 |  | 0.601 |  |
| Many worries or often seems worried | 8 |  | 0.540 |  | 0.610 |  |
| Often unhappy, depressed or tearful | 13 |  | 0.744 |  | 0.815 |  |
| Nervous in new situations, easily loses confidence | 16 |  | 0.719 |  | 0.644 |  |
| Many fears, easily scared | 24 |  | 0.642 |  | 0.679 |  |
| Often loses temper | 5 |  |  | 0.765 |  | 0.754 |
| Generally well behaved, usually does what adults request | 7 |  |  | 0.672 |  | 0.199 |
| Often fights with other youth or bullies them | 12 |  |  | 0.724 |  | 0.568 |
| Often lies or cheats | 18 |  |  | 0.728 |  | 0.520 |
| Steals from home, school or elsewhere | 22 |  |  | 0.615 |  | 0.279 |
| **Model fit (standard)** |  |  |  |  |  |  |
| **SRMR** |  |  | 0.078 | | 0.064 | |
| **RMSEA** |  |  | 0.022 | | 0.017 | |
| **RMSEA 90% CI** |  |  | [0.021, 0.023] | | [0.016, 0.018] | |
| **CFI** |  |  | 0.959 | | 0.957 | |
| **TLI** |  |  | 0.952 | | 0.950 | |
| **Reliability** |  |  |  |  |  |  |
| **ω** |  |  | 0.685 | 0.728 | 0.727 | 0.481 |

Note: Factor loading and model fit results of confirmatory factor analysis of SDQ (outcome). Int: Internalising. Ext: Externalising. SRMR: Standardized root mean square residuals; RMSEA: Root Mean Square Error of Approximation; CI: Confidence interval. CFI: Comparative Fit Index; TLI: Tucker-Lewis Index

| **Table S3 - Measurement invariance of parent-report SDQ across study type** | | | | | | | | | | | | | |
| --- | --- | --- | --- | --- | --- | --- | --- | --- | --- | --- | --- | --- | --- |
| Sample sizes | Invariance | χ2 | *df* | RMSEA | CFI | TLI | SRMR | Model comparison | Δχ2 (Δdf) | Δ CFI | Δ RMSEA | Δ SRMR | Decision |
| BHRCS=2,010 MCS = 10,126 | Config | 2632.52 | 328 | 0.037 | 0.97 | 0.969 | 0.078 |  |  |  |  |  |  |
|  | Metric | 2839.80 | 344 | 0.037 | 0.97 | 0.968 | 0.078 | Configural | 364.2672 (16) | 0.002 | 0.001 | 0.000 | Accept |
|  | Scalar | 3342.88 | 360 | 0.040 | 0.96 | 0.963 | 0.090 | Metriiic | 290.5075 (16) | 0.006 | 0.003 | 0.011 | Accept |
| Note: Invariance decision is based on ΔCFI < 0.010 supplemented by ΔRMSEA < 0.015 or ΔSRMR < 0.010. χ2, Chi square test; DF, degrees of freedom; RMSEA, Root Mean Square Error of Approximation; CFI, Comparative Fit Index; TLI, Tucker-Lewis Index; SRMR, Standardized Root Mean-square Residual; Δ, differences between fit index. **, p < 0.01; ***, p < 0.001 | | | | | | | | | | | | | |

| **Table S4 - Measurement invariance of self-report SDQ across study type** | | | | | | | | | | | | | |
| --- | --- | --- | --- | --- | --- | --- | --- | --- | --- | --- | --- | --- | --- |
| Sample sizes | Invariance | χ2 | *df* | RMSEA | CFI | TLI | SRMR | Model comparison | Δχ2 (Δdf) | Δ CFI | Δ RMSEA | Δ SRMR | Decision |
| BHRCS = 1,711 MCS = 9,733 | Configural | 2589.47 | 328 | 0.036 | 0.952 | 0.945 | 0.066 |  |  |  |  |  |  |
|  | Metric | 3168.65 | 344 | 0.039 | 0.940 | 0.934 | 0.066 | Configural | 689.1647 (16) | 0.012 | 0.003 | 0.000 | Accept |
|  | Scalar | 3612.47 | 360 | 0.041 | 0.931 | 0.927 | 0.080 | Metric | 231.5215 (16) | 0.009 | 0.002 | 0.013 | Accept |
| Note: Invariance decision is based on ΔCFI < 0.010 supplemented by ΔRMSEA < 0.015 or ΔSRMR < 0.010. χ2, Chi square test; DF, degrees of freedom; RMSEA, Root Mean Square Error of Approximation; CFI, Comparative Fit Index; TLI, Tucker-Lewis Index; SRMR, Standardized Root Mean-square Residual; Δ, differences between fit index. **, p < 0.01; ***, p < 0.001 | | | | | | | | | | | | | |

| **Table S5 – Pooled regression analysis: number of people living in household** | | | | | | | |
| --- | --- | --- | --- | --- | --- | --- | --- |
|  | **Internalising** | | |  | **Externalising** | | |
| **Characteristic** | **Beta** | **95% CI** | **p-value*** |  | **Beta** | **95% CI** | **p-value*** |
| Number of people living in household | 0.016 | 0.003, 0.029 | 0.052 |  | -0.019 | -0.035, -0.004 | 0.041 |
| Number of people living in household*Study (MCS:BHRCS) | -0.051 | -0.078, -0.023 | 0.001 |  | 0.018 | -0.014, 0.049 | 0.412 |
| **Covariates** |  |  |  |  |  |  |  |
| Study: MCS | 0.221 | 0.096, 0.345 | <0.001 |  | -0.559 | -0.703, -0.415 | <0.001 |
| prior mental health SDQ: Parent-report | 0.254 | 0.234, 0.273 | <0.001 |  | 0.4 | 0.377, 0.422 | <0.001 |
| Sex: female | 0.481 | 0.449, 0.512 | <0.001 |  | 0.188 | 0.151, 0.224 | <0.001 |
| Ethnicity: minority | -0.059 | -0.094, -0.024 | <0.001 |  | -0.079 | -0.119, -0.038 | <0.001 |
| Maternal education: complete secondary degree | -0.06 | -0.092, -0.028 | <0.001 |  | -0.076 | -0.114, -0.039 | <0.001 |
| Location: urban | -0.023 | -0.089, 0.043 | 0.497 |  | -0.005 | -0.081, 0.072 | 0.904 |
| Age in years | 0.002 | -0.008, 0.012 | 0.667 |  | -0.006 | -0.017, 0.005 | 0.296 |
| N | 9,142 |  |  |  | 9,142 |  |  |
| R2 | 0.162 |  |  |  | 0.170 |  |  |
| Note: CI = Confidence Interval; *, adjusted for false discovery rate with Benjamini-Hochberg method | | | | | | | |

| **Table S6 - Pooled regression analysis: siblings living in household** | | | | | | | |
| --- | --- | --- | --- | --- | --- | --- | --- |
|  | **Internalising** | | |  | **Externalising** | | |
| **Characteristic** | **Beta** | **95% CI** | **p-value*** |  | **Beta** | **95% CI** | **p-value*** |
| Siblings living in household | -0.03 | -0.067, 0.008 | 0.237 |  | -0.018 | -0.062, 0.026 | 0.519 |
| Siblings living in household*Study (MCS:BHRCS) | -0.061 | -0.152, 0.029 | 0.307 |  | -0.001 | -0.106, 0.104 | 0.986 |
| **Covariates** |  |  |  |  |  |  |  |
| Study: MCS | 0.065 | -0.020, 0.149 | 0.132 |  | -0.48 | -0.578, -0.382 | <0.001 |
| prior mental health SDQ: Parent-report | 0.254 | 0.234, 0.274 | <0.001 |  | 0.398 | 0.376, 0.420 | <0.001 |
| Sex: female | 0.482 | 0.451, 0.514 | <0.001 |  | 0.189 | 0.153, 0.225 | <0.001 |
| Ethnicity: minority | -0.058 | -0.093, -0.023 | 0.001 |  | -0.084 | -0.124, -0.044 | <0.001 |
| Maternal education: complete secondary degree | -0.063 | -0.095, -0.031 | <0.001 |  | -0.071 | -0.108, -0.034 | <0.001 |
| Location: urban | -0.024 | -0.090, 0.042 | 0.472 |  | -0.003 | -0.080, 0.073 | 0.935 |
| Age in years | 0.003 | -0.007, 0.013 | 0.581 |  | -0.005 | -0.016, 0.007 | 0.419 |
| N | 9,145 |  |  |  | 9,145 |  |  |
| R2 | 0.161 |  |  |  | 0.17 |  |  |
| Note: CI = Confidence Interval; *, adjusted for false discovery rate with Benjamini-Hochberg method | | | |  |  |  |  |

| **Table S7 - Pooled regression analysis: halfsiblings living in household** | | | | | | |  |
| --- | --- | --- | --- | --- | --- | --- | --- |
|  | **Internalising** | | |  | **Externalising** | | |
| **Characteristic** | **Beta** | **95% CI** | **p-value*** |  | **Beta** | **95% CI** | **p-value*** |
| Halfsiblings living in household | 0.119 | 0.076, 0.163 | <0.001 |  | 0.057 | 0.006, 0.107 | 0.063 |
| Halfsiblings living in household*Study (MCS:BHRCS) | -0.021 | -0.119, 0.076 | 0.799 |  | 0.197 | 0.084, 0.310 | 0.003 |
| **Covariates** |  |  |  |  |  |  |  |
| Study: MCS | 0.017 | -0.026, 0.060 | 0.435 |  | -0.506 | -0.556, -0.456 | <0.001 |
| prior mental health SDQ: Parent-report | 0.25 | 0.231, 0.270 | <0.001 |  | 0.391 | 0.368, 0.413 | <0.001 |
| Sex: female | 0.48 | 0.448, 0.511 | <0.001 |  | 0.187 | 0.151, 0.223 | <0.001 |
| Ethnicity: minority | -0.061 | -0.096, -0.026 | <0.001 |  | -0.082 | -0.123, -0.042 | <0.001 |
| Maternal education: complete secondary degree | -0.049 | -0.082, -0.017 | 0.003 |  | -0.06 | -0.097, -0.022 | 0.002 |
| Location: urban | -0.025 | -0.091, 0.040 | 0.448 |  | -0.008 | -0.084, 0.068 | 0.836 |
| Age in years | 0.003 | -0.007, 0.012 | 0.591 |  | -0.005 | -0.016, 0.006 | 0.390 |
| N | 9,145 |  |  |  | 9,145 |  |  |
| R2 | 0.164 |  |  |  | 0.172 |  |  |
| Note: CI = Confidence Interval; *, adjusted for false discovery rate with Benjamini-Hochberg method | | | |  |  |  |  |

| **Table S8 - Pooled regression analysis: poor quality of maternal-child relationship (get along in Brazil, closeness in UK)** | | | | | | |  |
| --- | --- | --- | --- | --- | --- | --- | --- |
|  | **Internalising** | | |  | **Externalising** | | |
| **Characteristic** | **Beta** | **95% CI** | **p-value*** |  | **Beta** | **95% CI** | **p-value*** |
| Poor quality of maternal-child relationship | 0.031 | 0.003, 0.060 | 0.078 |  | 0.016 | -0.017, 0.050 | 0.454 |
| Poor quality of maternal-child relationship*Study (MCS:BHRCS) | 0.005 | -0.045, 0.055 | 0.941 |  | 0.083 | 0.025, 0.140 | 0.018 |
| **Covariates** |  |  |  |  |  |  |  |
| Study: MCS | 0.002 | -0.051, 0.055 | 0.929 |  | -0.541 | -0.602, -0.480 | <0.001 |
| prior mental health SDQ: Parent-report | 0.251 | 0.231, 0.271 | <0.001 |  | 0.389 | 0.366, 0.412 | <0.001 |
| Sex: female | 0.48 | 0.449, 0.512 | <0.001 |  | 0.187 | 0.151, 0.224 | <0.001 |
| Ethnicity: minority | -0.058 | -0.094, -0.023 | 0.001 |  | -0.08 | -0.121, -0.040 | <0.001 |
| Maternal education: complete secondary degree | -0.062 | -0.094, -0.030 | <0.001 |  | -0.072 | -0.109, -0.035 | <0.001 |
| Location: urban | -0.024 | -0.090, 0.043 | 0.485 |  | -0.008 | -0.085, 0.069 | 0.843 |
| Age in years | 0.001 | -0.009, 0.011 | 0.853 |  | -0.005 | -0.017, 0.006 | 0.351 |
| N | 9,014 |  |  |  | 9,014 |  |  |
| R2 | 0.161 |  |  |  | 0.172 |  |  |
| Note: CI = Confidence Interval; *, adjusted for false discovery rate with Benjamini-Hochberg method | | | | | | | |

| **Table S9 - Pooled regression analysis: maternal marital status** | | | |  |  |  |  |
| --- | --- | --- | --- | --- | --- | --- | --- |
|  | **Internalising** | | |  | **Externalising** | | |
| **Characteristic** | **Beta** | **95% CI** | **p-value*** |  | **Beta** | **95% CI** | **p-value*** |
| Separated and living with someone else | 0.141 | 0.088, 0.195 | <0.001 |  | 0.094 | 0.032, 0.156 | 0.013 |
| Single | 0.088 | 0.036, 0.140 | 0.003 |  | 0.045 | -0.015, 0.105 | 0.253 |
| Separated/divorced | 0.103 | 0.047, 0.158 | 0.001 |  | 0.145 | 0.080, 0.209 | <0.001 |
| Widow | 0.084 | -0.028, 0.195 | 0.249 |  | 0.105 | -0.024, 0.235 | 0.218 |
| Separated and living with someone else*Study (MCS:BHRCS) | -0.101 | -0.220, 0.018 | 0.205 |  | -0.081 | -0.218, 0.057 | 0.397 |
| Single*Study (MCS:BHRCS) | 0.036 | -0.060, 0.132 | 0.601 |  | 0.145 | 0.034, 0.256 | 0.035 |
| Separated/divorced*Study (MCS:BHRCS) | -0.018 | -0.118, 0.082 | 0.838 |  | 0.055 | -0.061, 0.171 | 0.46 |
| Widow*Study (MCS:BHRCS) | -0.098 | -0.404, 0.208 | 0.661 |  | -0.052 | -0.406, 0.302 | 0.859 |
| **Covariates** |  |  |  |  |  |  |  |
| Study: MCS | 0.019 | -0.031, 0.070 | 0.453 |  | -0.505 | -0.564, -0.446 | <0.001 |
| prior mental health SDQ: Parent-report | 0.249 | 0.229, 0.269 | <0.001 |  | 0.391 | 0.369, 0.413 | <0.001 |
| Sex: female | 0.479 | 0.448, 0.511 | <0.001 |  | 0.183 | 0.147, 0.220 | <0.001 |
| Ethnicity: minority | -0.065 | -0.099, -0.030 | <0.001 |  | -0.086 | -0.126, -0.045 | <0.001 |
| Maternal education: complete secondary degree | -0.055 | -0.087, -0.023 | <0.001 |  | -0.062 | -0.099, -0.024 | 0.001 |
| Location: urban | -0.027 | -0.093, 0.039 | 0.423 |  | -0.013 | -0.090, 0.063 | 0.732 |
| Age in years | 0.002 | -0.008, 0.011 | 0.75 |  | -0.006 | -0.018, 0.005 | 0.267 |
| N | 9,140 |  |  |  | 9,140 |  |  |
| R2 | 0.165 |  |  |  | 0.175 |  |  |
| Note: CI = Confidence Interval; *, adjusted for false discovery rate with Benjamini-Hochberg method | | | | | | | |

| **Table S10 - Pooled regression analysis: paternal death** | | | | | |  |  |
| --- | --- | --- | --- | --- | --- | --- | --- |
|  | **Internalising** | | |  | **Externalising** | | |
| **Characteristic** | **Beta** | **95% CI** | **p-value*** |  | **Beta** | **95% CI** | **p-value*** |
| Paternal death | -0.003 | -0.088, 0.082 | 0.942 |  | 0.001 | -0.097, 0.099 | 0.986 |
| Paternal death*Study (MCS:BHRCS) | 0.019 | -0.310, 0.347 | 0.942 |  | 0.305 | -0.076, 0.686 | 0.218 |
| **Covariates** |  |  |  |  |  |  |  |
| Study: MCS | 0.003 | -0.038, 0.044 | 0.894 |  | -0.489 | -0.536, -0.441 | <0.001 |
| prior mental health SDQ: Parent-report | 0.254 | 0.234, 0.274 | <0.001 |  | 0.398 | 0.376, 0.420 | <0.001 |
| Sex: female | 0.482 | 0.450, 0.513 | <0.001 |  | 0.189 | 0.152, 0.225 | <0.001 |
| Ethnicity: minority | -0.059 | -0.094, -0.024 | <0.001 |  | -0.084 | -0.125, -0.044 | <0.001 |
| Maternal education: complete secondary degree | -0.062 | -0.095, -0.030 | <0.001 |  | -0.07 | -0.108, -0.033 | <0.001 |
| Location: urban | -0.025 | -0.090, 0.041 | 0.465 |  | -0.003 | -0.080, 0.073 | 0.932 |
| Age in years | 0.002 | -0.008, 0.012 | 0.693 |  | -0.005 | -0.016, 0.006 | 0.368 |
| N | 9,145 |  |  |  | 9,145 |  |  |
| R2 | 0.160 |  |  |  | 0.170 |  |  |
| Note: CI = Confidence Interval; *, adjusted for false discovery rate with Benjamini-Hochberg method | | | | | | | |

| **Table S11 - Pooled regression analysis: difficulty in getting along or keeping friends** | | | | | |  |  |
| --- | --- | --- | --- | --- | --- | --- | --- |
|  | **Internalising** | | |  | **Externalising** | | |
| **Characteristic** | **Beta** | **95% CI** | **p-value*** |  | **Beta** | **95% CI** | **p-value*** |
| Difficulty in getting along or keeping friends | 0.164 | 0.087, 0.241 | <0.001 |  | 0.102 | 0.013, 0.190 | 0.06 |
| Difficulty in getting along or keeping friends*Study (MCS:BHRCS) | -0.057 | -0.155, 0.042 | 0.411 |  | 0.105 | -0.008, 0.218 | 0.149 |
| **Covariates** |  |  |  |  |  |  |  |
| Study: MCS | -0.006 | -0.054, 0.041 | 0.79 |  | -0.553 | -0.608, -0.498 | <0.001 |
| prior mental health SDQ: Parent-report | 0.246 | 0.226, 0.267 | <0.001 |  | 0.386 | 0.363, 0.408 | <0.001 |
| Sex: female | 0.487 | 0.455, 0.519 | <0.001 |  | 0.196 | 0.159, 0.232 | <0.001 |
| Ethnicity: minority | -0.045 | -0.081, -0.010 | 0.012 |  | -0.07 | -0.111, -0.029 | <0.001 |
| Maternal education: complete secondary degree | -0.065 | -0.098, -0.032 | <0.001 |  | -0.067 | -0.104, -0.029 | <0.001 |
| Location: urban | -0.025 | -0.093, 0.043 | 0.472 |  | -0.022 | -0.101, 0.056 | 0.577 |
| Age in years | 0.003 | -0.007, 0.013 | 0.565 |  | -0.005 | -0.016, 0.007 | 0.409 |
| N | 8,760 |  |  |  | 8,760 |  |  |
| R2 | 0.167 |  |  |  | 0.174 |  |  |
| Note: CI = Confidence Interval; *, adjusted for false discovery rate with Benjamini-Hochberg method | | | | | | | |

| **Table S12 - Pooled regression analysis: done bullying** | | | | | | | |  |  |
| --- | --- | --- | --- | --- | --- | --- | --- | --- | --- |
|  | **Internalising** | | |  | | **Externalising** | | | |
| **Characteristic** | **Beta** | **95% CI** | **p-value*** | |  | | **Beta** | **95% CI** | **p-value*** |
| Have you ever done bullying | 0.112 | 0.054, 0.170 | 0.001 | |  | | 0.189 | 0.122, 0.257 | <0.001 |
| Have you ever done bullying*Study (MCS:BHRCS) | 0.092 | 0.007, 0.176 | 0.079 | |  | | 0.292 | 0.194, 0.390 | <0.001 |
| **Covariates** |  |  |  | |  | |  |  |  |
| Study: MCS | -0.05 | -0.095, -0.005 | 0.03 | |  | | -0.611 | -0.663, -0.559 | <0.001 |
| prior mental health SDQ: Parent-report | 0.256 | 0.236, 0.276 | <0.001 | |  | | 0.382 | 0.359, 0.404 | <0.001 |
| Sex: female | 0.512 | 0.480, 0.544 | <0.001 | |  | | 0.226 | 0.189, 0.262 | <0.001 |
| Ethnicity: minority | -0.064 | -0.100, -0.029 | <0.001 | |  | | -0.086 | -0.127, -0.045 | <0.001 |
| Maternal education: complete secondary degree | -0.054 | -0.087, -0.022 | 0.001 | |  | | -0.051 | -0.089, -0.014 | 0.007 |
| Location: urban | -0.031 | -0.095, 0.034 | 0.35 | |  | | -0.006 | -0.080, 0.069 | 0.883 |
| Age in years | 0.001 | -0.009, 0.011 | 0.817 | |  | | -0.011 | -0.022, 0.001 | 0.068 |
| N | 8,837 |  |  | |  | | 8,837 |  |  |
| R2 | 0.176 |  |  | |  | | 0.192 |  |  |
| Note: CI = Confidence Interval; *, adjusted for false discovery rate with Benjamini-Hochberg method | | | | | | | | | |

| **Table S13 - Pooled regression analysis: been bullied** | | | | |  | |  |  |  |
| --- | --- | --- | --- | --- | --- | --- | --- | --- | --- |
|  | **Internalising** | | |  | | **Externalising** | | | |
| **Characteristic** | **Beta** | **95% CI** | **p-value*** | |  | | **Beta** | **95% CI** | **p-value*** |
| Have you ever been bullied | 0.21 | 0.169, 0.252 | <0.001 | |  | | 0.169 | 0.120, 0.217 | <0.001 |
| Have you ever been bullied*Study (MCS:BHRCS) | 0.148 | 0.079, 0.217 | <0.001 | |  | | 0.21 | 0.129, 0.291 | <0.001 |
| **Covariates** |  |  |  | |  | |  |  |  |
| Study: MCS | -0.12 | -0.171, -0.070 | <0.001 | |  | | -0.634 | -0.692, -0.575 | <0.001 |
| prior mental health SDQ: Parent-report | 0.245 | 0.225, 0.265 | <0.001 | |  | | 0.385 | 0.363, 0.408 | <0.001 |
| Sex: female | 0.502 | 0.470, 0.533 | <0.001 | |  | | 0.2 | 0.164, 0.237 | <0.001 |
| Ethnicity: minority | -0.052 | -0.087, -0.016 | 0.004 | |  | | -0.072 | -0.113, -0.030 | <0.001 |
| Maternal education: complete secondary degree | -0.053 | -0.085, -0.021 | 0.001 | |  | | -0.05 | -0.088, -0.013 | 0.009 |
| Location: urban | -0.01 | -0.074, 0.054 | 0.752 | |  | | 0.018 | -0.057, 0.093 | 0.637 |
| Age in years | 0.003 | -0.006, 0.013 | 0.496 | |  | | -0.007 | -0.018, 0.005 | 0.244 |
| N | 8,840 |  |  | |  | | 8,840 |  |  |
| R2 | 0.192 |  |  | |  | | 0.190 |  |  |
| Note: CI = Confidence Interval; *, adjusted for false discovery rate with Benjamini-Hochberg method | | | | | | | | | |

| **Table S14 - Pooled regression analysis: has at least one good friend, parent-report** | | | | | | | | | |  |
| --- | --- | --- | --- | --- | --- | --- | --- | --- | --- | --- |
|  | **Internalising** | | | |  | | **Externalising** | | | |
| **Characteristic** | **Beta** | **95% CI** | **p-value*** |  | | **Beta** | | **95% CI** | **p-value*** | |
| The child does not have at least one good friend | 0.031 | -0.032, 0.094 | 0.480 |  | | 0.003 | | -0.070, 0.076 | 0.986 | |
| The child does not have at least one good friend*Study (MCS:BHRCS) | -0.01 | -0.186, 0.167 | 0.942 |  | | 0.072 | | -0.132, 0.276 | 0.584 | |
| **Covariates** |  |  |  |  | |  | |  |  | |
| Study: MCS | 0.005 | -0.037, 0.047 | 0.819 |  | | -0.489 | | -0.538, -0.441 | <0.001 | |
| prior mental health SDQ: Parent-report | 0.252 | 0.231, 0.272 | <0.001 |  | | 0.396 | | 0.373, 0.419 | <0.001 | |
| Sex: female | 0.483 | 0.452, 0.515 | <0.001 |  | | 0.189 | | 0.153, 0.225 | <0.001 | |
| Ethnicity: minority | -0.061 | -0.096, -0.026 | <0.001 |  | | -0.086 | | -0.126, -0.045 | <0.001 | |
| Maternal education: complete secondary degree | -0.062 | -0.094, -0.030 | <0.001 |  | | -0.071 | | -0.109, -0.034 | <0.001 | |
| Location: urban | -0.024 | -0.090, 0.042 | 0.481 |  | | -0.005 | | -0.082, 0.071 | 0.89 | |
| Age in years | 0.002 | -0.008, 0.012 | 0.706 |  | | -0.005 | | -0.016, 0.006 | 0.364 | |
| N | 9,104 |  |  |  | | 9,104 | |  |  | |
| R2 | 0.161 |  |  |  | | 0.169 | |  |  | |
| Note: CI = Confidence Interval; *, adjusted for false discovery rate with Benjamini-Hochberg method | | | | | | | | | | |

| **Table S15 - Pooled regression analysis: has at least one good friend, self-report** | | | | | | | | |  |
| --- | --- | --- | --- | --- | --- | --- | --- | --- | --- |
|  | **Internalising** | | |  | | **Externalising** | | | |
| **Characteristic** | **Beta** | **95% CI** | **p-value*** | |  | | **Beta** | **95% CI** | **p-value*** |
| I do not have at least one good friend | 0.032 | -0.046, 0.110 | 0.578 | |  | | 0.048 | -0.043, 0.139 | 0.426 |
| I do not have at least one good friend*Study (MCS:BHRCS) | 0.101 | -0.085, 0.287 | 0.433 | |  | | 0.066 | -0.150, 0.282 | 0.633 |
| **Covariates** |  |  |  | |  | |  |  |  |
| Study: MCS | 0.017 | -0.025, 0.058 | 0.427 | |  | | -0.48 | -0.528, -0.432 | <0.001 |
| prior mental health SDQ: Parent-report | 0.253 | 0.233, 0.273 | <0.001 | |  | | 0.396 | 0.374, 0.419 | <0.001 |
| Sex: female | 0.492 | 0.460, 0.524 | <0.001 | |  | | 0.186 | 0.149, 0.223 | <0.001 |
| Ethnicity: minority | -0.054 | -0.090, -0.019 | 0.003 | |  | | -0.095 | -0.136, -0.054 | <0.001 |
| Maternal education: complete secondary degree | -0.045 | -0.078, -0.013 | 0.006 | |  | | -0.057 | -0.095, -0.020 | 0.003 |
| Location: urban | -0.025 | -0.090, 0.040 | 0.453 | |  | | 0.001 | -0.075, 0.076 | 0.982 |
| Age in years | 0.004 | -0.006, 0.014 | 0.38 | |  | | -0.002 | -0.014, 0.009 | 0.683 |
| N | 8,943 |  |  | |  | | 8,943 |  |  |
| R2 | 0.162 |  |  | |  | | 0.167 |  |  |
| Note: CI = Confidence Interval; *, adjusted for false discovery rate with Benjamini-Hochberg method | | | | | | | | | |

| **Table S16 - Pooled regression analysis: moved to a different house or city since last interview** | | | | | | |  |
| --- | --- | --- | --- | --- | --- | --- | --- |
|  | **Internalising** | | |  | **Externalising** | | |
| **Characteristic** | **Beta** | **95% CI** | **p-value*** |  | **Beta** | **95% CI** | **p-value*** |
| Moved to a different house or city since last interview | 0.081 | 0.037, 0.124 | 0.001 |  | 0.062 | 0.012, 0.112 | 0.041 |
| Moved to a different house or city since last interview*Study (MCS:BHRCS) | -0.072 | -0.161, 0.017 | 0.224 |  | 0.072 | -0.031, 0.175 | 0.284 |
| **Covariates** |  |  |  |  |  |  |  |
| Study: MCS | 0.022 | -0.022, 0.066 | 0.32 |  | -0.492 | -0.543, -0.441 | <0.001 |
| prior mental health SDQ: Parent-report | 0.253 | 0.233, 0.273 | <0.001 |  | 0.396 | 0.373, 0.418 | <0.001 |
| Sex: female | 0.483 | 0.452, 0.515 | <0.001 |  | 0.189 | 0.153, 0.225 | <0.001 |
| Ethnicity: minority | -0.057 | -0.092, -0.023 | 0.001 |  | -0.082 | -0.123, -0.042 | <0.001 |
| Maternal education: complete secondary degree | -0.067 | -0.100, -0.035 | <0.001 |  | -0.072 | -0.110, -0.035 | <0.001 |
| Location: urban | -0.025 | -0.091, 0.041 | 0.458 |  | -0.003 | -0.079, 0.074 | 0.944 |
| Age in years | 0.004 | -0.006, 0.013 | 0.455 |  | -0.004 | -0.015, 0.007 | 0.503 |
| N | 9,145 |  |  |  | 9,145 |  |  |
| R2 | 0.162 |  |  |  | 0.171 |  |  |
| Note: CI = Confidence Interval; *, adjusted for false discovery rate with Benjamini-Hochberg method | | | | | | | |

| **Table S17 - BHRCS regression analysis: number of people living in household** | | | | | | | |
| --- | --- | --- | --- | --- | --- | --- | --- |
|  | **Internalising** | | |  | **Externalising** | | |
| **Characteristic** | **Beta** | **95% CI** | **p-value*** |  | **Beta** | **95% CI** | **p-value*** |
| Number of people living in household | 0.014 | -0.014, 0.041 | 0.501 |  | -0.016 | -0.044, 0.011 | 0.445 |
| **Covariates** |  |  |  |  |  |  |  |
| SDQ factor score: Parent-report | 0.246 | 0.196, 0.296 | <0.001 |  | 0.334 | 0.285, 0.382 | <0.001 |
| Sex: female | 0.517 | 0.438, 0.595 | <0.001 |  | 0.322 | 0.244, 0.399 | <0.001 |
| Ethnicity: minority | -0.037 | -0.116, 0.042 | 0.36 |  | -0.069 | -0.147, 0.009 | 0.083 |
| Maternal education: complete secondary degree | -0.093 | -0.173, -0.013 | 0.023 |  | -0.09 | -0.168, -0.011 | 0.026 |
| Age in years | 0.002 | -0.018, 0.023 | 0.834 |  | -0.008 | -0.029, 0.012 | 0.415 |
| N | 1,473 |  |  |  | 1,473 |  |  |
| R2 | 0.169 |  |  |  | 0.147 |  |  |
| Note: CI = Confidence Interval; *, adjusted for false discovery rate with Benjamini-Hochberg method | | | | | | | |

| **Table S18 - BHRCS regression analysis: siblings living in household** | | | | | | | |
| --- | --- | --- | --- | --- | --- | --- | --- |
|  | **Internalising** | | |  | **Externalising** | | |
| **Characteristic** | **Beta** | **95% CI** | **p-value*** |  | **Beta** | **95% CI** | **p-value*** |
| Siblings living in household | -0.033 | -0.112, 0.045 | 0.553 |  | -0.023 | -0.101, 0.055 | 0.607 |
| **Covariates** |  |  |  |  |  |  |  |
| SDQ factor score: Parent-report | 0.247 | 0.197, 0.297 | <0.001 |  | 0.332 | 0.283, 0.380 | <0.001 |
| Sex: female | 0.519 | 0.440, 0.597 | <0.001 |  | 0.324 | 0.247, 0.401 | <0.001 |
| Ethnicity: minority | -0.034 | -0.112, 0.045 | 0.403 |  | -0.074 | -0.151, 0.004 | 0.063 |
| Maternal education: complete secondary degree | -0.098 | -0.177, -0.019 | 0.015 |  | -0.084 | -0.162, -0.005 | 0.036 |
| Age in years | 0.003 | -0.018, 0.024 | 0.775 |  | -0.007 | -0.027, 0.013 | 0.496 |
| N | 1,476 |  |  |  | 1,476 |  |  |
| R2 | 0.169 |  |  |  | 0.146 |  |  |
| Note: CI = Confidence Interval; *, adjusted for false discovery rate with Benjamini-Hochberg method | | | | | | | |

| **Table S19 - BHRCS regression analysis: halfsiblings living in household** | | | | | | | |
| --- | --- | --- | --- | --- | --- | --- | --- |
|  | **Internalising** | | |  | **Externalising** | | |
| **Characteristic** | **Beta** | **95% CI** | **p-value*** |  | **Beta** | **95% CI** | **p-value*** |
| Halfsiblings living in household | 0.112 | 0.020, 0.203 | 0.086 |  | 0.059 | -0.032, 0.149 | 0.436 |
| **Covariates** |  |  |  |  |  |  |  |
| SDQ factor score: Parent-report | 0.244 | 0.194, 0.293 | <0.001 |  | 0.328 | 0.280, 0.377 | <0.001 |
| Sex: female | 0.515 | 0.436, 0.593 | <0.001 |  | 0.321 | 0.244, 0.398 | <0.001 |
| Ethnicity: minority | -0.037 | -0.116, 0.041 | 0.351 |  | -0.076 | -0.153, 0.002 | 0.056 |
| Maternal education: complete secondary degree | -0.083 | -0.162, -0.003 | 0.043 |  | -0.075 | -0.154, 0.004 | 0.062 |
| Age in years | 0.003 | -0.018, 0.023 | 0.786 |  | -0.007 | -0.027, 0.013 | 0.478 |
| N | 1,476 |  |  |  | 1,476 |  |  |
| R2 | 0.172 |  |  |  | 0.147 |  |  |
| Note: CI = Confidence Interval; *, adjusted for false discovery rate with Benjamini-Hochberg method | | | | | | | |

| **Table S20 - BHRCS regression analysis: poor quality of maternal-child relationship (get along in Brazil, closeness in UK)** | | | | | | | |
| --- | --- | --- | --- | --- | --- | --- | --- |
|  | **Internalising** | | |  | **Externalising** | | |
| **Characteristic** | **Beta** | **95% CI** | **p-value*** |  | **Beta** | **95% CI** | **p-value*** |
| Poor quality of maternal-child relationship | 0.029 | -0.030, 0.089 | 0.501 |  | 0.025 | -0.034, 0.085 | 0.576 |
| **Covariates** |  |  |  |  |  |  |  |
| SDQ factor score: Parent-report | 0.244 | 0.194, 0.294 | <0.001 |  | 0.327 | 0.278, 0.377 | <0.001 |
| Sex: female | 0.516 | 0.437, 0.594 | <0.001 |  | 0.319 | 0.241, 0.396 | <0.001 |
| Ethnicity: minority | -0.034 | -0.113, 0.044 | 0.391 |  | -0.072 | -0.150, 0.006 | 0.069 |
| Maternal education: complete secondary degree | -0.094 | -0.174, -0.015 | 0.02 |  | -0.081 | -0.159, -0.003 | 0.042 |
| Age in years | 0.001 | -0.019, 0.022 | 0.906 |  | -0.008 | -0.028, 0.012 | 0.45 |
| N | 1,474 |  |  |  | 1,474 |  |  |
| R2 | 0.169 |  |  |  | 0.146 |  |  |
| Note: CI = Confidence Interval; *, adjusted for false discovery rate with Benjamini-Hochberg method | | | | | | | |

| **Table S21 - BHRCS regression analysis: maternal marital status** | | | | | | | | |
| --- | --- | --- | --- | --- | --- | --- | --- | --- |
|  | **Internalising** | | |  | **Externalising** | | | |
| **Characteristic** | **Beta** | **95% CI** | **p-value*** |  | **Beta** | **95% CI** | **p-value*** |  |
| Separated and living with someone else | 0.136 | 0.024, 0.249 | 0.086 |  | 0.093 | -0.018, 0.204 | 0.276 |  |
| Single | 0.084 | -0.025, 0.193 | 0.243 |  | 0.044 | -0.063, 0.151 | 0.576 |  |
| Separated/divorced | 0.097 | -0.018, 0.213 | 0.213 |  | 0.126 | 0.011, 0.240 | 0.158 |  |
| Widow | 0.079 | -0.154, 0.311 | 0.633 |  | 0.098 | -0.132, 0.328 | 0.576 |  |
| **Covariates** |  |  |  |  |  |  |  |  |
| SDQ factor score: Parent-report | 0.244 | 0.194, 0.294 | <0.001 |  | 0.33 | 0.281, 0.379 | <0.001 |  |
| Sex: female | 0.514 | 0.435, 0.593 | <0.001 |  | 0.317 | 0.240, 0.395 | <0.001 |  |
| Ethnicity: minority | -0.042 | -0.121, 0.037 | 0.295 |  | -0.079 | -0.157, -0.002 | 0.046 |  |
| Maternal education: complete secondary degree | -0.092 | -0.172, -0.013 | 0.023 |  | -0.081 | -0.159, -0.002 | 0.044 |  |
| Age in years | 0.002 | -0.019, 0.022 | 0.87 |  | -0.009 | -0.029, 0.012 | 0.397 |  |
| N | 1,473 |  |  |  | 1,473 |  |  |  |
| R2 | 0.173 |  |  |  | 0.15 |  |  |  |
| Note: CI = Confidence Interval; *, adjusted for false discovery rate with Benjamini-Hochberg method | | | | | | | | |

| **Table S22 - BHRCS regression analysis: paternal death** | | | | | | | |
| --- | --- | --- | --- | --- | --- | --- | --- |
|  | **Internalising** | | |  | **Externalising** | | |
| **Characteristic** | **Beta** | **95% CI** | **p-value*** |  | **Beta** | **95% CI** | **p-value*** |
| Paternal death | -0.01 | -0.188, 0.167 | 0.908 |  | 0.003 | -0.172, 0.178 | 0.974 |
| **Covariates** |  |  |  |  |  |  |  |
| SDQ factor score: Parent-report | 0.247 | 0.197, 0.297 | <0.001 |  | 0.331 | 0.283, 0.380 | <0.001 |
| Sex: female | 0.518 | 0.439, 0.596 | <0.001 |  | 0.323 | 0.246, 0.400 | <0.001 |
| Ethnicity: minority | -0.034 | -0.113, 0.044 | 0.392 |  | -0.074 | -0.152, 0.003 | 0.061 |
| Maternal education: complete secondary degree | -0.097 | -0.177, -0.018 | 0.017 |  | -0.082 | -0.161, -0.004 | 0.039 |
| Age in years | 0.002 | -0.018, 0.023 | 0.841 |  | -0.008 | -0.028, 0.013 | 0.457 |
| N | 1,476 |  |  |  | 1,476 |  |  |
| R2 | 0.169 |  |  |  | 0.146 |  |  |
| Note: CI = Confidence Interval; *, adjusted for false discovery rate with Benjamini-Hochberg method | | | | | | | |

| **Table S23 - BHRCS regression analysis: difficulty in getting along or keeping friends** | | | | | | | |
| --- | --- | --- | --- | --- | --- | --- | --- |
|  | **Internalising** | | |  | **Externalising** | | |
| **Characteristic** | **Beta** | **95% CI** | **p-value*** |  | **Beta** | **95% CI** | **p-value*** |
| Difficulty in getting along or keeping friends | 0.168 | 0.008, 0.327 | 0.148 |  | 0.133 | -0.025, 0.291 | 0.276 |
| **Covariates** |  |  |  |  |  |  |  |
| SDQ factor score: Parent-report | 0.238 | 0.187, 0.288 | <0.001 |  | 0.325 | 0.275, 0.374 | <0.001 |
| Sex: female | 0.519 | 0.441, 0.597 | <0.001 |  | 0.323 | 0.245, 0.400 | <0.001 |
| Ethnicity: minority | -0.025 | -0.104, 0.054 | 0.534 |  | -0.065 | -0.143, 0.013 | 0.1 |
| Maternal education: complete secondary degree | -0.095 | -0.174, -0.016 | 0.018 |  | -0.081 | -0.159, -0.003 | 0.042 |
| Age in years | 0.003 | -0.018, 0.023 | 0.801 |  | -0.007 | -0.027, 0.013 | 0.486 |
| N | 1,472 |  |  |  | 1,472 |  |  |
| R2 | 0.172 |  |  |  | 0.149 |  |  |
| Note: CI = Confidence Interval; *, adjusted for false discovery rate with Benjamini-Hochberg method | | | | | | | |

| **Table S24 - BHRCS regression analysis: done bullying** | | | | | | | |
| --- | --- | --- | --- | --- | --- | --- | --- |
|  | **Internalising** | | |  | **Externalising** | | |
| **Characteristic** | **Beta** | **95% CI** | **p-value*** |  | **Beta** | **95% CI** | **p-value*** |
| Have you ever done bullying | 0.115 | -0.009, 0.240 | 0.173 |  | 0.227 | 0.105, 0.349 | 0.002 |
| **Covariates** |  |  |  |  |  |  |  |
| SDQ factor score: Parent-report | 0.252 | 0.199, 0.304 | <0.001 |  | 0.318 | 0.267, 0.368 | <0.001 |
| Sex: female | 0.555 | 0.472, 0.638 | <0.001 |  | 0.369 | 0.288, 0.450 | <0.001 |
| Ethnicity: minority | -0.041 | -0.123, 0.042 | 0.336 |  | -0.083 | -0.164, -0.002 | 0.046 |
| Maternal education: complete secondary degree | -0.083 | -0.166, 0.001 | 0.052 |  | -0.045 | -0.126, 0.037 | 0.285 |
| Age in years | 0.002 | -0.020, 0.023 | 0.889 |  | -0.013 | -0.034, 0.009 | 0.246 |
| N | 1,324 |  |  |  | 1,324 |  |  |
| R2 | 0.186 |  |  |  | 0.165 |  |  |
| Note: CI = Confidence Interval; *, adjusted for false discovery rate with Benjamini-Hochberg method | | | | | | | |

| **Table S25 - BHRCS regression analysis: been bullied** | | | | | | | |
| --- | --- | --- | --- | --- | --- | --- | --- |
|  | **Internalising** | | |  | **Externalising** | | |
| **Characteristic** | **Beta** | **95% CI** | **p-value*** |  | **Beta** | **95% CI** | **p-value*** |
| Have you ever been bullied | 0.209 | 0.120, 0.298 | <0.001 |  | 0.178 | 0.090, 0.266 | 0.001 |
| **Covariates** |  |  |  |  |  |  |  |
| SDQ factor score: Parent-report | 0.245 | 0.193, 0.298 | <0.001 |  | 0.321 | 0.270, 0.371 | <0.001 |
| Sex: female | 0.545 | 0.463, 0.627 | <0.001 |  | 0.349 | 0.269, 0.429 | <0.001 |
| Ethnicity: minority | -0.033 | -0.115, 0.049 | 0.429 |  | -0.074 | -0.154, 0.007 | 0.074 |
| Maternal education: complete secondary degree | -0.075 | -0.158, 0.007 | 0.074 |  | -0.044 | -0.125, 0.037 | 0.29 |
| Age in years | 0.004 | -0.018, 0.025 | 0.722 |  | -0.008 | -0.029, 0.013 | 0.437 |
| N | 1,330 |  |  |  | 1,330 |  |  |
| R2 | 0.195 |  |  |  | 0.166 |  |  |
| Note: CI = Confidence Interval; *, adjusted for false discovery rate with Benjamini-Hochberg method | | | | | | | |

| **Table S26 - BHRCS regression analysis: has at least one good friend, parent-report** | | | | | | | |
| --- | --- | --- | --- | --- | --- | --- | --- |
|  | **Internalising** | | |  | **Externalising** | | |
| **Characteristic** | **Beta** | **95% CI** | **p-value*** |  | **Beta** | **95% CI** | **p-value*** |
| The child does not have at least one good friend | 0.034 | -0.099, 0.168 | 0.706 |  | 0.048 | -0.083, 0.179 | 0.588 |
| **Covariates** |  |  |  |  |  |  |  |
| SDQ factor score: Parent-report | 0.243 | 0.192, 0.295 | <0.001 |  | 0.327 | 0.277, 0.377 | <0.001 |
| Sex: female | 0.518 | 0.440, 0.597 | <0.001 |  | 0.323 | 0.246, 0.400 | <0.001 |
| Ethnicity: minority | -0.035 | -0.114, 0.044 | 0.383 |  | -0.075 | -0.152, 0.003 | 0.059 |
| Maternal education: complete secondary degree | -0.096 | -0.176, -0.017 | 0.017 |  | -0.082 | -0.160, -0.004 | 0.04 |
| Age in years | 0.002 | -0.018, 0.022 | 0.85 |  | -0.008 | -0.028, 0.012 | 0.449 |
| N | 1,476 |  |  |  | 1,476 |  |  |
| R2 | 0.169 |  |  |  | 0.146 |  |  |
| Note: CI = Confidence Interval; *, adjusted for false discovery rate with Benjamini-Hochberg method | | | | | | | |

| **Table S27 - BHRCS regression analysis: has at least one good friend, self-report** | | | | | | | |
| --- | --- | --- | --- | --- | --- | --- | --- |
|  | **Internalising** | | |  | **Externalising** | | |
| **Characteristic** | **Beta** | **95% CI** | **p-value*** |  | **Beta** | **95% CI** | **p-value*** |
| I do not have at least one good friend | 0.032 | -0.135, 0.199 | 0.758 |  | 0.055 | -0.109, 0.220 | 0.588 |
| **Covariates** |  |  |  |  |  |  |  |
| SDQ factor score: Parent-report | 0.245 | 0.192, 0.298 | <0.001 |  | 0.328 | 0.277, 0.379 | <0.001 |
| Sex: female | 0.535 | 0.452, 0.617 | <0.001 |  | 0.324 | 0.243, 0.404 | <0.001 |
| Ethnicity: minority | -0.027 | -0.109, 0.056 | 0.528 |  | -0.086 | -0.167, -0.005 | 0.038 |
| Maternal education: complete secondary degree | -0.074 | -0.157, 0.009 | 0.082 |  | -0.064 | -0.145, 0.017 | 0.122 |
| Age in years | 0.005 | -0.017, 0.026 | 0.664 |  | -0.005 | -0.026, 0.016 | 0.654 |
| N | 1,351 |  |  |  | 1,351 |  |  |
| R2 | 0.17 |  |  |  | 0.145 |  |  |
| Note: CI = Confidence Interval; *, adjusted for false discovery rate with Benjamini-Hochberg method | | | | | | | |

| **Table S28 - BHRCS regression analysis: moved to a different house or city since last interview** | | | | | | | | | | |
| --- | --- | --- | --- | --- | --- | --- | --- | --- | --- | --- |
|  | **Internalising** | | | |  | | **Externalising** | | | |
| **Characteristic** | **Beta** | **95% CI** | **p-value*** |  | | **Beta** | | **95% CI** | **p-value*** |  |
| Moved to a different house or city since last interview | 0.088 | -0.002, 0.179 | 0.166 |  | | 0.073 | | -0.017, 0.162 | 0.276 |  |
| **Covariates** |  |  |  |  | |  | |  |  |  |
| SDQ factor score: Parent-report | 0.245 | 0.195, 0.295 | <0.001 |  | | 0.33 | | 0.282, 0.379 | <0.001 |  |
| Sex: female | 0.52 | 0.442, 0.598 | <0.001 |  | | 0.324 | | 0.247, 0.402 | <0.001 |  |
| Ethnicity: minority | -0.032 | -0.111, 0.046 | 0.42 |  | | -0.072 | | -0.150, 0.005 | 0.067 |  |
| Maternal education: complete secondary degree | -0.105 | -0.184, -0.025 | 0.01 |  | | -0.089 | | -0.167, -0.011 | 0.026 |  |
| Age in years | 0.004 | -0.017, 0.025 | 0.704 |  | | -0.006 | | -0.026, 0.014 | 0.557 |  |
| N | 1,476 |  |  |  | | 1,476 | |  |  |  |
| R2 | 0.171 |  |  |  | | 0.148 | |  |  |  |
| Note: CI = Confidence Interval; *, adjusted for false discovery rate with Benjamini-Hochberg method | | | | | | | | | | |

| **Table S29 - MCS regression analysis: number of people in household** | | | | | | | |
| --- | --- | --- | --- | --- | --- | --- | --- |
|  | **Internalising** | | |  | **Externalising** | | |
| **Characteristic** | **Beta** | **95% CI** | **p-value*** |  | **Beta** | **95% CI** | **p-value*** |
| Number of people living in household | -0.029 | -0.045, -0.012 | 0.002 |  | -0.013 | -0.037, 0.011 | 0.381 |
| **Covariates** |  |  |  |  |  |  |  |
| SDQ factor score: Parent-report | 0.265 | 0.241, 0.289 | <0.001 |  | 0.512 | 0.479, 0.545 | <0.001 |
| Sex: female | 0.409 | 0.369, 0.448 | <0.001 |  | -0.082 | -0.140, -0.024 | 0.005 |
| Ethnicity: minority | -0.198 | -0.255, -0.141 | <0.001 |  | -0.136 | -0.220, -0.052 | 0.001 |
| Maternal education: complete secondary degree | 0.036 | -0.005, 0.077 | 0.086 |  | 0.034 | -0.027, 0.094 | 0.273 |
| Age in years | -0.083 | -0.140, -0.026 | 0.005 |  | -0.095 | -0.178, -0.011 | 0.027 |
| N | 5,809 |  |  |  | 5,809 |  |  |
| R2 | 0.152 |  |  |  | 0.144 |  |  |
| Note: CI = Confidence Interval; *, adjusted for false discovery rate with Benjamini-Hochberg method | | | | | | | |

| **Table S30 - MCS regression analysis: siblings living in household** | | | | | | | |
| --- | --- | --- | --- | --- | --- | --- | --- |
|  | **Internalising** | | |  | **Externalising** | | |
| **Characteristic** | **Beta** | **95% CI** | **p-value*** |  | **Beta** | **95% CI** | **p-value*** |
| Siblings living in household | -0.106 | -0.163, -0.049 | 0.001 |  | -0.04 | -0.123, 0.043 | 0.401 |
| **Covariates** |  |  |  |  |  |  |  |
| SDQ factor score: Parent-report | 0.264 | 0.239, 0.288 | <0.001 |  | 0.512 | 0.478, 0.545 | <0.001 |
| Sex: female | 0.407 | 0.368, 0.447 | <0.001 |  | -0.082 | -0.140, -0.025 | 0.005 |
| Ethnicity: minority | -0.213 | -0.269, -0.157 | <0.001 |  | -0.143 | -0.225, -0.060 | <0.001 |
| Maternal education: complete secondary degree | 0.041 | 0.000, 0.082 | 0.048 |  | 0.036 | -0.024, 0.096 | 0.242 |
| Age in years | -0.083 | -0.140, -0.026 | 0.004 |  | -0.095 | -0.178, -0.011 | 0.027 |
| N | 5,809 |  |  |  | 5,809 |  |  |
| R2 | 0.152 |  |  |  | 0.144 |  |  |
| Note: CI = Confidence Interval; *, adjusted for false discovery rate with Benjamini-Hochberg method | | | | | | | |

| **Table S31 - MCS regression analysis: halfsiblings living in household** | | | | | | | |
| --- | --- | --- | --- | --- | --- | --- | --- |
|  | **Internalising** | | |  | **Externalising** | | |
| **Characteristic** | **Beta** | **95% CI** | **p-value*** |  | **Beta** | **95% CI** | **p-value*** |
| Halfsiblings living in household | 0.079 | 0.020, 0.139 | 0.015 |  | 0.177 | 0.089, 0.265 | <0.001 |
| **Covariates** |  |  |  |  |  |  |  |
| SDQ factor score: Parent-report | 0.262 | 0.238, 0.287 | <0.001 |  | 0.500 | 0.467, 0.534 | <0.001 |
| Sex: female | 0.409 | 0.370, 0.449 | <0.001 |  | -0.084 | -0.141, -0.026 | 0.005 |
| Ethnicity: minority | -0.21 | -0.267, -0.154 | <0.001 |  | -0.13 | -0.212, -0.047 | 0.002 |
| Maternal education: complete secondary degree | 0.045 | 0.005, 0.086 | 0.03 |  | 0.046 | -0.015, 0.106 | 0.137 |
| Age in years | -0.086 | -0.143, -0.028 | 0.003 |  | -0.101 | -0.185, -0.017 | 0.018 |
| N | 5,809 |  |  |  | 5,809 |  |  |
| R2 | 0.151 |  |  |  | 0.146 |  |  |
| Note: CI = Confidence Interval; *, adjusted for false discovery rate with Benjamini-Hochberg method | | | | | | | |

| **Table S32 - MCS regression analysis: poor quality of maternal-child relationship (get along in Brazil, closeness in UK)** | | | | | | | |  |
| --- | --- | --- | --- | --- | --- | --- | --- | --- |
|  | **Internalising** | | |  | **Externalising** | | | |
| **Characteristic** | **Beta** | **95% CI** | **p-value*** |  | **Beta** | **95% CI** | **p-value*** | |
| Poor quality of maternal-child relationship | 0.052 | 0.024, 0.080 | 0.001 |  | 0.062 | 0.020, 0.104 | 0.007 | |
| **Covariates** |  |  |  |  |  |  |  | |
| SDQ factor score: Parent-report | 0.261 | 0.236, 0.285 | <0.001 |  | 0.499 | 0.465, 0.534 | <0.001 | |
| Sex: female | 0.408 | 0.369, 0.448 | <0.001 |  | -0.084 | -0.142, -0.026 | 0.005 | |
| Ethnicity: minority | -0.221 | -0.279, -0.163 | <0.001 |  | -0.133 | -0.218, -0.048 | 0.002 | |
| Maternal education: complete secondary degree | 0.037 | -0.004, 0.078 | 0.079 |  | 0.031 | -0.031, 0.092 | 0.328 | |
| Age in years | -0.094 | -0.152, -0.036 | 0.001 |  | -0.106 | -0.191, -0.021 | 0.015 | |
| N | 5,689 |  |  |  | 5,689 |  |  | |
| R2 | 0.152 |  |  |  | 0.145 |  |  | |
| Note: CI = Confidence Interval; *, adjusted for false discovery rate with Benjamini-Hochberg method | | | | | | | |  |

| **Table S33 - MCS regression analysis: maternal marital status** | | | | | | | |
| --- | --- | --- | --- | --- | --- | --- | --- |
|  | **Internalising** | | |  | **Externalising** | | |
| **Characteristic** | **Beta** | **95% CI** | **p-value*** |  | **Beta** | **95% CI** | **p-value*** |
| Separated and living with someone else | 0.072 | -0.003, 0.147 | 0.092 |  | 0.029 | -0.081, 0.139 | 0.644 |
| Single | 0.089 | 0.033, 0.146 | 0.004 |  | 0.123 | 0.041, 0.205 | 0.007 |
| Separated/divorced | 0.08 | 0.023, 0.136 | 0.01 |  | 0.171 | 0.089, 0.254 | <0.001 |
| Widow | -0.055 | -0.254, 0.143 | 0.585 |  | -0.152 | -0.442, 0.138 | 0.381 |
| **Covariates** |  |  |  |  |  |  |  |
| SDQ factor score: Parent-report | 0.259 | 0.234, 0.283 | <0.001 |  | 0.496 | 0.463, 0.530 | <0.001 |
| Sex: female | 0.409 | 0.370, 0.449 | <0.001 |  | -0.083 | -0.141, -0.026 | 0.005 |
| Ethnicity: minority | -0.206 | -0.262, -0.149 | <0.001 |  | -0.127 | -0.210, -0.045 | 0.003 |
| Maternal education: complete secondary degree | 0.05 | 0.009, 0.091 | 0.017 |  | 0.054 | -0.007, 0.115 | 0.08 |
| Age in years | -0.085 | -0.142, -0.028 | 0.004 |  | -0.092 | -0.176, -0.008 | 0.031 |
| N | 5,807 |  |  |  | 5,807 |  |  |
| R2 | 0.152 |  |  |  | 0.147 |  |  |
| Note: CI = Confidence Interval; *, adjusted for false discovery rate with Benjamini-Hochberg method | | | | | | | |

| **Table S34 - MCS regression analysis: paternal death** | | | | | | | |
| --- | --- | --- | --- | --- | --- | --- | --- |
|  | **Internalising** | | |  | **Externalising** | | |
| **Characteristic** | **Beta** | **95% CI** | **p-value*** |  | **Beta** | **95% CI** | **p-value*** |
| Paternal death | 0.062 | -0.154, 0.279 | 0.585 |  | 0.181 | -0.136, 0.498 | 0.381 |
| **Covariates** |  |  |  |  |  |  |  |
| SDQ factor score: Parent-report | 0.266 | 0.242, 0.291 | <0.001 |  | 0.511 | 0.478, 0.545 | <0.001 |
| Sex: female | 0.409 | 0.370, 0.448 | <0.001 |  | -0.081 | -0.139, -0.023 | 0.006 |
| Ethnicity: minority | -0.216 | -0.273, -0.160 | <0.001 |  | -0.144 | -0.227, -0.062 | <0.001 |
| Maternal education: complete secondary degree | 0.04 | -0.001, 0.081 | 0.053 |  | 0.036 | -0.024, 0.096 | 0.24 |
| Age in years | -0.083 | -0.140, -0.025 | 0.005 |  | -0.095 | -0.179, -0.011 | 0.027 |
| N | 5,809 |  |  |  | 5,809 |  |  |
| R2 | 0.15 |  |  |  | 0.144 |  |  |
| Note: CI = Confidence Interval; *, adjusted for false discovery rate with Benjamini-Hochberg method | | | | | | | |

| **Table S35 - MCS regression analysis: difficulty in getting along or keeping friends** | | | | | | | |
| --- | --- | --- | --- | --- | --- | --- | --- |
|  | **Internalising** | | |  | **Externalising** | | |
| **Characteristic** | **Beta** | **95% CI** | **p-value*** |  | **Beta** | **95% CI** | **p-value*** |
| Difficulty in getting along or keeping friends | 0.109 | 0.067, 0.152 | <0.001 |  | 0.213 | 0.150, 0.276 | <0.001 |
| **Covariates** |  |  |  |  |  |  |  |
| SDQ factor score: Parent-report | 0.262 | 0.237, 0.287 | <0.001 |  | 0.488 | 0.454, 0.523 | <0.001 |
| Sex: female | 0.421 | 0.381, 0.462 | <0.001 |  | -0.079 | -0.138, -0.019 | 0.01 |
| Ethnicity: minority | -0.192 | -0.251, -0.133 | <0.001 |  | -0.106 | -0.193, -0.019 | 0.017 |
| Maternal education: complete secondary degree | 0.036 | -0.006, 0.079 | 0.092 |  | 0.051 | -0.011, 0.114 | 0.107 |
| Age in years | -0.049 | -0.108, 0.011 | 0.11 |  | -0.091 | -0.178, -0.004 | 0.041 |
| N | 5,489 |  |  |  | 5,489 |  |  |
| R2 | 0.158 |  |  |  | 0.145 |  |  |
| Note: CI = Confidence Interval; *, adjusted for false discovery rate with Benjamini-Hochberg method | | | | | | | |

| **Table S36 - MCS regression analysis: done bullying** | | | | | | | |
| --- | --- | --- | --- | --- | --- | --- | --- |
|  | **Internalising** | | |  | **Externalising** | | |
| **Characteristic** | **Beta** | **95% CI** | **p-value*** |  | **Beta** | **95% CI** | **p-value*** |
| Have you ever done bullying | 0.197 | 0.153, 0.241 | <0.001 |  | 0.433 | 0.369, 0.497 | <0.001 |
| **Covariates** |  |  |  |  |  |  |  |
| SDQ factor score: Parent-report | 0.261 | 0.236, 0.285 | <0.001 |  | 0.481 | 0.447, 0.515 | <0.001 |
| Sex: female | 0.429 | 0.389, 0.468 | <0.001 |  | -0.04 | -0.098, 0.018 | 0.173 |
| Ethnicity: minority | -0.206 | -0.262, -0.150 | <0.001 |  | -0.137 | -0.219, -0.055 | 0.001 |
| Maternal education: complete secondary degree | 0.027 | -0.015, 0.068 | 0.206 |  | 0.009 | -0.051, 0.069 | 0.78 |
| Age in years | -0.078 | -0.136, -0.021 | 0.008 |  | -0.081 | -0.164, 0.003 | 0.058 |
| N | 5,681 |  |  |  | 5,681 |  |  |
| R2 | 0.16 |  |  |  | 0.163 |  |  |
| Note: CI = Confidence Interval; *, adjusted for false discovery rate with Benjamini-Hochberg method | | | | | | | |

| **Table S37 - MCS regression analysis: been bullied** | | | | | | | |
| --- | --- | --- | --- | --- | --- | --- | --- |
|  | **Internalising** | | |  | **Externalising** | | |
| **Characteristic** | **Beta** | **95% CI** | **p-value*** |  | **Beta** | **95% CI** | **p-value*** |
| Have you ever been bullied | 0.356 | 0.317, 0.395 | <0.001 |  | 0.343 | 0.285, 0.401 | <0.001 |
| **Covariates** |  |  |  |  |  |  |  |
| SDQ factor score: Parent-report | 0.239 | 0.215, 0.264 | <0.001 |  | 0.489 | 0.455, 0.523 | <0.001 |
| Sex: female | 0.415 | 0.376, 0.453 | <0.001 |  | -0.08 | -0.138, -0.023 | 0.006 |
| Ethnicity: minority | -0.162 | -0.218, -0.107 | <0.001 |  | -0.096 | -0.178, -0.013 | 0.023 |
| Maternal education: complete secondary degree | 0.007 | -0.034, 0.047 | 0.746 |  | 0.008 | -0.052, 0.069 | 0.783 |
| Age in years | -0.066 | -0.123, -0.009 | 0.022 |  | -0.077 | -0.161, 0.006 | 0.071 |
| N | 5,675 |  |  |  | 5,675 |  |  |
| R2 | 0.194 |  |  |  | 0.158 |  |  |
| Note: CI = Confidence Interval; *, adjusted for false discovery rate with Benjamini-Hochberg method | | | | | | | |

| **Table S38 - MCS regression analysis: has at least one good friend, parent-report** | | | | | | | |
| --- | --- | --- | --- | --- | --- | --- | --- |
|  | **Internalising** | | |  | **Externalising** | | |
| **Characteristic** | **Beta** | **95% CI** | **p-value*** |  | **Beta** | **95% CI** | **p-value*** |
| The child does not have at least one good friend | 0.035 | -0.076, 0.146 | 0.585 |  | -0.094 | -0.257, 0.068 | 0.381 |
| **Covariates** |  |  |  |  |  |  |  |
| SDQ factor score: Parent-report | 0.264 | 0.238, 0.289 | <0.001 |  | 0.514 | 0.480, 0.548 | <0.001 |
| Sex: female | 0.414 | 0.374, 0.453 | <0.001 |  | -0.082 | -0.140, -0.024 | 0.006 |
| Ethnicity: minority | -0.23 | -0.287, -0.174 | <0.001 |  | -0.149 | -0.232, -0.066 | <0.001 |
| Maternal education: complete secondary degree | 0.039 | -0.001, 0.080 | 0.059 |  | 0.032 | -0.028, 0.093 | 0.296 |
| Age in years | -0.078 | -0.135, -0.020 | 0.008 |  | -0.092 | -0.176, -0.008 | 0.033 |
| N | 5,775 |  |  |  | 5,775 |  |  |
| R2 | 0.152 |  |  |  | 0.143 |  |  |
| Note: CI = Confidence Interval; *, adjusted for false discovery rate with Benjamini-Hochberg method | | | | | | | |

| **Table S39 - MCS regression analysis: has at least one good friend, self-report** | | | | | | | |
| --- | --- | --- | --- | --- | --- | --- | --- |
|  | **Internalising** | | |  | **Externalising** | | |
| **Characteristic** | **Beta** | **95% CI** | **p-value*** |  | **Beta** | **95% CI** | **p-value*** |
| I do not have at least one good friend | 0.085 | -0.031, 0.201 | 0.189 |  | -0.008 | -0.176, 0.161 | 0.930 |
| **Covariates** |  |  |  |  |  |  |  |
| SDQ factor score: Parent-report | 0.266 | 0.241, 0.291 | <0.001 |  | 0.511 | 0.477, 0.545 | <0.001 |
| Sex: female | 0.408 | 0.368, 0.448 | <0.001 |  | -0.077 | -0.135, -0.019 | 0.01 |
| Ethnicity: minority | -0.211 | -0.267, -0.154 | <0.001 |  | -0.134 | -0.217, -0.051 | 0.001 |
| Maternal education: complete secondary degree | 0.039 | -0.002, 0.080 | 0.064 |  | 0.034 | -0.026, 0.095 | 0.267 |
| Age in years | -0.077 | -0.134, -0.019 | 0.009 |  | -0.096 | -0.180, -0.012 | 0.025 |
| N | 5,743 |  |  |  | 5,743 |  |  |
| R2 | 0.15 |  |  |  | 0.141 |  |  |
| Note: CI = Confidence Interval; *, adjusted for false discovery rate with Benjamini-Hochberg method | | | | | | | |

| **Table S40 - MCS regression analysis: moved to a different house or city since last interview** | | | | | | | |
| --- | --- | --- | --- | --- | --- | --- | --- |
|  | **Internalising** | | |  | **Externalising** | | |
| **Characteristic** | **Beta** | **95% CI** | **p-value*** |  | **Beta** | **95% CI** | **p-value*** |
| Moved to a different house or city since last interview | 0.049 | -0.005, 0.103 | 0.105 |  | 0.155 | 0.076, 0.234 | <0.001 |
| **Covariates** |  |  |  |  |  |  |  |
| SDQ factor score: Parent-report | 0.265 | 0.241, 0.289 | <0.001 |  | 0.506 | 0.473, 0.539 | <0.001 |
| Sex: female | 0.408 | 0.369, 0.448 | <0.001 |  | -0.085 | -0.142, -0.027 | 0.004 |
| Ethnicity: minority | -0.215 | -0.272, -0.159 | <0.001 |  | -0.141 | -0.223, -0.058 | <0.001 |
| Maternal education: complete secondary degree | 0.043 | 0.002, 0.084 | 0.041 |  | 0.043 | -0.017, 0.103 | 0.162 |
| Age in years | -0.085 | -0.142, -0.028 | 0.004 |  | -0.101 | -0.185, -0.017 | 0.018 |
| N | 5,809 |  |  |  | 5,809 |  |  |
| R2 | 0.15 |  |  |  | 0.146 |  |  |
| Note: CI = Confidence Interval; *, adjusted for false discovery rate with Benjamini-Hochberg method | | | | | | | |

| **Table S41 - Unadjusted regression analysis: number of people living in household** | | | | | | | |
| --- | --- | --- | --- | --- | --- | --- | --- |
|  | **Internalising** | | |  | **Externalising** | | |
| **Characteristic** | **Beta** | **95% CI** | **p-value*** |  | **Beta** | **95% CI** | **p-value*** |
| Number of people living in household | 0.022 | 0.009, 0.036 | 0.003 |  | 0.003 | -0.013, 0.019 | 0.725 |
| Number of people living in household*Study (MCS:BHRCS) | -0.07 | -0.096, -0.045 | <0.001 |  | -0.017 | -0.047, 0.013 | 0.345 |
| Study:MCS | 0.349 | 0.233, 0.465 | <0.001 |  | -0.356 | -0.491, -0.221 | <0.001 |
| N | 10,599 |  |  |  | 10,599 |  |  |
| R2 | 0.003 |  |  |  | 0.043 |  |  |
| Note: CI = Confidence Interval; *, adjusted for false discovery rate with Benjamini-Hochberg method | | | | | | | |

| **Table S42 - Unadjusted regression analysis: siblings living in household** | | | | | | | |
| --- | --- | --- | --- | --- | --- | --- | --- |
|  | **Internalising** | | |  | **Externalising** | | |
| **Characteristic** | **Beta** | **95% CI** | **p-value*** |  | **Beta** | **95% CI** | **p-value*** |
| Siblings living in household | 0.005 | -0.034, 0.044 | 0.92 |  | -0.01 | -0.055, 0.035 | 0.725 |
| Siblings living in household*Study (MCS:BHRCS) | -0.151 | -0.238, -0.065 | 0.002 |  | -0.092 | -0.193, 0.008 | 0.108 |
| Study:MCS | 0.175 | 0.098, 0.252 | <0.001 |  | -0.347 | -0.436, -0.257 | <0.001 |
| N | 10,602 |  |  |  | 10,602 |  |  |
| R2 | 0.002 |  |  |  | 0.043 |  |  |
| Note: CI = Confidence Interval; *, adjusted for false discovery rate with Benjamini-Hochberg method | | | | | | | |

| **Table S43 - Unadjusted regression analysis: halfsiblings living in household** | | | | | | | |
| --- | --- | --- | --- | --- | --- | --- | --- |
|  | **Internalising** | | |  | **Externalising** | | |
| **Characteristic** | **Beta** | **95% CI** | **p-value*** |  | **Beta** | **95% CI** | **p-value*** |
| Halfsiblings living in household | 0.177 | 0.132, 0.222 | <0.001 |  | 0.14 | 0.088, 0.192 | <0.001 |
| Halfsiblings living in household*Study (MCS:BHRCS) | 0.009 | -0.084, 0.102 | 0.92 |  | 0.238 | 0.130, 0.346 | <0.001 |
| Study:MCS | 0.068 | 0.032, 0.105 | <0.001 |  | -0.441 | -0.484, -0.399 | <0.001 |
| N | 10,602 |  |  |  | 10,602 |  |  |
| R2 | 0.008 |  |  |  | 0.05 |  |  |
| Note: CI = Confidence Interval; *, adjusted for false discovery rate with Benjamini-Hochberg method | | | | | | | |

| **Table S44 - Unadjusted regression analysis: poor quality of maternal-child relationship (get along in Brazil, closeness in UK)** | | | | | | | |
| --- | --- | --- | --- | --- | --- | --- | --- |
|  | **Internalising** | | |  | **Externalising** | | |
| **Characteristic** | **Beta** | **95% CI** | **p-value*** |  | **Beta** | **95% CI** | **p-value*** |
| Poor quality of maternal-child relationship | 0.079 | 0.050, 0.109 | <0.001 |  | 0.115 | 0.081, 0.149 | <0.001 |
| Poor quality of maternal-child relationship*Study (MCS:BHRCS) | -0.024 | -0.075, 0.026 | 0.468 |  | 0.098 | 0.041, 0.156 | 0.002 |
| Study:MCS | 0.062 | 0.013, 0.110 | 0.012 |  | -0.515 | -0.571, -0.460 | <0.001 |
| N | 9,942 |  |  |  | 9,942 |  |  |
| R2 | 0.004 |  |  |  | 0.058 |  |  |
| Note: CI = Confidence Interval; *, adjusted for false discovery rate with Benjamini-Hochberg method | | | | | | | |

| **Table S45 - Unadjusted regression analysis: maternal marital status** | | | | | | | |
| --- | --- | --- | --- | --- | --- | --- | --- |
|  | **Internalising** | | |  | **Externalising** | | |
| **Characteristic** | **Beta** | **95% CI** | **p-value*** |  | **Beta** | **95% CI** | **p-value*** |
| Separated and living with someone else | 0.171 | 0.115, 0.227 | <0.001 |  | 0.151 | 0.086, 0.215 | <0.001 |
| Single | 0.126 | 0.071, 0.180 | <0.001 |  | 0.087 | 0.024, 0.150 | 0.013 |
| Separated/divorced | 0.158 | 0.100, 0.216 | <0.001 |  | 0.149 | 0.083, 0.216 | <0.001 |
| Widow | 0.078 | -0.039, 0.196 | 0.321 |  | 0.056 | -0.079, 0.191 | 0.502 |
| Separated and living with someone else*Study (MCS:BHRCS) | -0.06 | -0.179, 0.058 | 0.454 |  | -0.024 | -0.161, 0.112 | 0.725 |
| Single*Study (MCS:BHRCS) | 0.092 | -0.006, 0.190 | 0.115 |  | 0.235 | 0.123, 0.347 | <0.001 |
| Separated/divorced*Study (MCS:BHRCS) | 0.035 | -0.066, 0.136 | 0.646 |  | 0.224 | 0.108, 0.340 | <0.001 |
| Widow*Study (MCS:BHRCS) | -0.029 | -0.345, 0.288 | 0.92 |  | 0.08 | -0.284, 0.444 | 0.725 |
| Study:MCS | 0.048 | 0.002, 0.094 | 0.041 |  | -0.501 | -0.554, -0.448 | <0.001 |
| N | 10,087 |  |  |  | 10,087 |  |  |
| R2 | 0.01 |  |  |  | 0.058 |  |  |
| Note: CI = Confidence Interval; *, adjusted for false discovery rate with Benjamini-Hochberg method | | | | | | | |

| **Table S46 - Unadjusted regression analysis: paternal death** | | | | | | | |
| --- | --- | --- | --- | --- | --- | --- | --- |
|  | **Internalising** | | |  | **Externalising** | | |
| **Characteristic** | **Beta** | **95% CI** | **p-value*** |  | **Beta** | **95% CI** | **p-value*** |
| Paternal death | -0.019 | -0.108, 0.069 | 0.805 |  | 0.018 | -0.084, 0.121 | 0.725 |
| Paternal death*Study (MCS:BHRCS) | 0.017 | -0.300, 0.333 | 0.95 |  | 0.288 | -0.079, 0.655 | 0.170 |
| Study:MCS | 0.046 | 0.012, 0.080 | 0.007 |  | -0.431 | -0.470, -0.391 | <0.001 |
| N | 10,602 |  |  |  | 10,602 |  |  |
| R2 | 0.001 |  |  |  | 0.043 |  |  |
| Note: CI = Confidence Interval; *, adjusted for false discovery rate with Benjamini-Hochberg method | | | | | | | |

| **Table S47 - Unadjusted regression analysis: difficulty in getting along or keeping friends** | | | | | | | |
| --- | --- | --- | --- | --- | --- | --- | --- |
|  | **Internalising** | | |  | **Externalising** | | |
| **Characteristic** | **Beta** | **95% CI** | **p-value*** |  | **Beta** | **95% CI** | **p-value*** |
| Difficulty in getting along or keeping friends | 0.343 | 0.265, 0.422 | <0.001 |  | 0.364 | 0.274, 0.454 | <0.001 |
| Difficulty in getting along or keeping friends*Study (MCS:BHRCS) | -0.168 | -0.267, -0.069 | 0.002 |  | -0.061 | -0.174, 0.053 | 0.370 |
| Study:MCS | 0.02 | -0.020, 0.060 | 0.333 |  | -0.516 | -0.562, -0.469 | <0.001 |
| N | 10,103 |  |  |  | 10,103 |  |  |
| R2 | 0.011 |  |  |  | 0.056 |  |  |
| Note: CI = Confidence Interval; *, adjusted for false discovery rate with Benjamini-Hochberg method | | | | | | | |

| **Table S48 - Unadjusted regression analysis: done bullying** | | | | | | | |
| --- | --- | --- | --- | --- | --- | --- | --- |
|  | **Internalising** | | |  | **Externalising** | | |
| **Characteristic** | **Beta** | **95% CI** | **p-value*** |  | **Beta** | **95% CI** | **p-value*** |
| Have you ever done bullying | 0.093 | 0.033, 0.153 | 0.005 |  | 0.28 | 0.211, 0.350 | <0.001 |
| Have you ever done bullying*Study (MCS:BHRCS) | 0.044 | -0.041, 0.128 | 0.454 |  | 0.244 | 0.147, 0.342 | <0.001 |
| Study:MCS | 0.016 | -0.023, 0.054 | 0.424 |  | -0.557 | -0.601, -0.513 | <0.001 |
| N | 10,245 |  |  |  | 10,245 |  |  |
| R2 | 0.003 |  |  |  | 0.072 |  |  |
| Note: CI = Confidence Interval; *, adjusted for false discovery rate with Benjamini-Hochberg method | | | | | | | |

| **Table S49 - Unadjusted regression analysis: been bullied** | | | | | | | |  |
| --- | --- | --- | --- | --- | --- | --- | --- | --- |
|  | **Internalising** | | |  | **Externalising** | | | |
| **Characteristic** | **Beta** | **95% CI** | **p-value*** |  | **Beta** | **95% CI** | **p-value*** | |
| Have you ever been bullied | 0.257 | 0.213, 0.300 | <0.001 |  | 0.252 | 0.201, 0.302 | <0.001 | |
| Have you ever been bullied*Study (MCS:BHRCS) | 0.144 | 0.075, 0.212 | <0.001 |  | 0.199 | 0.119, 0.278 | <0.001 | |
| Study:MCS | -0.084 | -0.128, -0.039 | <0.001 |  | -0.598 | -0.650, -0.546 | <0.001 | |
| N | 10,247 |  |  |  | 10,247 |  |  | |
| R2 | 0.034 |  |  |  | 0.073 |  |  | |
| Note: CI = Confidence Interval; *, adjusted for false discovery rate with Benjamini-Hochberg method | | | | | | | |  |

| **Table S50 - Unadjusted regression analysis: has at least one good friend, parent-report** | | | | | | | |
| --- | --- | --- | --- | --- | --- | --- | --- |
|  | **Internalising** | | |  | **Externalising** | | |
| **Characteristic** | **Beta** | **95% CI** | **p-value*** |  | **Beta** | **95% CI** | **p-value*** |
| The child does not have at least one good friend | 0.171 | 0.105, 0.238 | <0.001 |  | 0.244 | 0.169, 0.320 | <0.001 |
| The child does not have at least one good friend*Study (MCS:BHRCS) | 0.042 | -0.147, 0.230 | 0.805 |  | 0.21 | -0.004, 0.425 | 0.087 |
| Study:MCS | 0.056 | 0.019, 0.094 | 0.003 |  | -0.435 | -0.477, -0.392 | <0.001 |
| N | 9,431 |  |  |  | 9,431 |  |  |
| R2 | 0.004 |  |  |  | 0.051 |  |  |
| Note: CI = Confidence Interval; *, adjusted for false discovery rate with Benjamini-Hochberg method | | | | | | | |

| **Table S51 - Unadjusted regression analysis: has at least one good friend, self-report** | | | | | | | |
| --- | --- | --- | --- | --- | --- | --- | --- |
|  | **Internalising** | | |  | **Externalising** | | |
| **Characteristic** | **Beta** | **95% CI** | **p-value*** |  | **Beta** | **95% CI** | **p-value*** |
| I do not have at least one good friend | 0.045 | -0.036, 0.126 | 0.435 |  | 0.074 | -0.020, 0.168 | 0.170 |
| I do not have at least one good friend*Study (MCS:BHRCS) | 0.177 | 0.002, 0.353 | 0.089 |  | 0.246 | 0.042, 0.450 | 0.030 |
| Study:MCS | 0.061 | 0.026, 0.095 | <0.001 |  | -0.423 | -0.463, -0.383 | <0.001 |
| N | 10,366 |  |  |  | 10,366 |  |  |
| R2 | 0.002 |  |  |  | 0.042 |  |  |
| Note: CI = Confidence Interval; *, adjusted for false discovery rate with Benjamini-Hochberg method | | | | | | | |

| **Table S52 - Unadjusted regression analysis: moved to a different house or city since last interview** | | | | | | | |
| --- | --- | --- | --- | --- | --- | --- | --- |
|  | **Internalising** | | |  | **Externalising** | | |
| **Characteristic** | **Beta** | **95% CI** | **p-value*** |  | **Beta** | **95% CI** | **p-value*** |
| Moved to a different house or city since last interview | 0.074 | 0.029, 0.119 | 0.003 |  | 0.064 | 0.012, 0.116 | 0.027 |
| Moved to a different house or city since last interview*Study (MCS:BHRCS) | -0.002 | -0.092, 0.087 | 0.957 |  | 0.154 | 0.051, 0.256 | 0.007 |
| Study:MCS | 0.053 | 0.014, 0.091 | 0.007 |  | -0.464 | -0.509, -0.420 | <0.001 |
| N | 10,111 |  |  |  | 10,111 |  |  |
| R2 | 0.002 |  |  |  | 0.048 |  |  |
| Note: CI = Confidence Interval; *, adjusted for false discovery rate with Benjamini-Hochberg method | | | | | | | |

| **Table S53 - Urban population regression analysis: number of people living in household** | | | | | | | |  |
| --- | --- | --- | --- | --- | --- | --- | --- | --- |
|  | **Internalising** | | |  | **Externalising** | | | |
| **Characteristic** | **Beta** | **95% CI** | **p-value*** |  | **Beta** | **95% CI** | **p-value*** | |
| Number of people living in household | 0.019 | -0.001, 0.039 | 0.151 |  | -0.02 | -0.046, 0.006 | 0.369 | |
| Number of people living in household*Study (MCS:BHRCS) | -0.054 | -0.081, -0.027 | 0.001 |  | 0.006 | -0.030, 0.041 | 0.868 | |
| **Covariates** |  |  |  |  |  |  |  | |
| Study: MCS | 0.215 | 0.092, 0.338 | <0.001 |  | -0.526 | -0.687, -0.365 | <0.001 | |
| SDQ factor score: Parent-report | 0.255 | 0.233, 0.277 | <0.001 |  | 0.451 | 0.423, 0.478 | <0.001 | |
| Sex: female | 0.453 | 0.418, 0.488 | <0.001 |  | 0.073 | 0.027, 0.119 | 0.002 | |
| Ethnicity: minority | -0.104 | -0.147, -0.061 | <0.001 |  | -0.098 | -0.154, -0.042 | <0.001 | |
| Maternal education: complete secondary degree | -0.02 | -0.056, 0.016 | 0.278 |  | -0.034 | -0.082, 0.013 | 0.159 | |
| Age in years | 0 | -0.015, 0.014 | 0.963 |  | -0.006 | -0.024, 0.013 | 0.559 | |
| N | 7,282 |  |  |  | 7,282 |  |  | |
| R2 | 0.154 |  |  |  | 0.162 |  |  | |
| Note: CI = Confidence Interval; *, adjusted for false discovery rate with Benjamini-Hochberg method | | | | | | | |  |

| **Table S54 - Urban population regression analysis: siblings living in household** | | | | | | | |
| --- | --- | --- | --- | --- | --- | --- | --- |
|  | **Internalising** | | |  | **Externalising** | | |
| **Characteristic** | **Beta** | **95% CI** | **p-value*** |  | **Beta** | **95% CI** | **p-value*** |
| Siblings living in household | -0.024 | -0.080, 0.033 | 0.631 |  | -0.011 | -0.086, 0.063 | 0.868 |
| Siblings living in household*Study (MCS:BHRCS) | -0.083 | -0.170, 0.004 | 0.151 |  | -0.02 | -0.133, 0.093 | 0.868 |
| **Covariates** |  |  |  |  |  |  |  |
| Study: MCS | 0.061 | -0.016, 0.138 | 0.12 |  | -0.484 | -0.584, -0.384 | <0.001 |
| SDQ factor score: Parent-report | 0.254 | 0.232, 0.276 | <0.001 |  | 0.45 | 0.422, 0.477 | <0.001 |
| Sex: female | 0.453 | 0.418, 0.488 | <0.001 |  | 0.074 | 0.028, 0.120 | 0.002 |
| Ethnicity: minority | -0.109 | -0.152, -0.067 | <0.001 |  | -0.105 | -0.160, -0.049 | <0.001 |
| Maternal education: complete secondary degree | -0.019 | -0.056, 0.017 | 0.29 |  | -0.03 | -0.077, 0.018 | 0.218 |
| Age in years | 0 | -0.014, 0.014 | 0.996 |  | -0.004 | -0.023, 0.014 | 0.641 |
| N | 7,285 |  |  |  | 7,285 |  |  |
| R2 | 0.154 |  |  |  | 0.162 |  |  |
| Note: CI = Confidence Interval; *, adjusted for false discovery rate with Benjamini-Hochberg method | | | | | | | |

| **Table S55 - Urban population regression analysis: halfsiblings living in household** | | | | | | | |
| --- | --- | --- | --- | --- | --- | --- | --- |
|  | **Internalising** | | |  | **Externalising** | | |
| **Characteristic** | **Beta** | **95% CI** | **p-value*** |  | **Beta** | **95% CI** | **p-value*** |
| Halfsiblings living in household | 0.129 | 0.064, 0.194 | 0.001 |  | 0.061 | -0.024, 0.146 | 0.370 |
| Halfsiblings living in household*Study (MCS:BHRCS) | -0.051 | -0.145, 0.043 | 0.493 |  | 0.132 | 0.010, 0.254 | 0.172 |
| **Covariates** |  |  |  |  |  |  |  |
| Study: MCS | 0.002 | -0.042, 0.047 | 0.915 |  | -0.513 | -0.571, -0.455 | <0.001 |
| SDQ factor score: Parent-report | 0.252 | 0.230, 0.274 | <0.001 |  | 0.44 | 0.413, 0.468 | <0.001 |
| Sex: female | 0.452 | 0.417, 0.488 | <0.001 |  | 0.072 | 0.026, 0.118 | 0.002 |
| Ethnicity: minority | -0.111 | -0.153, -0.068 | <0.001 |  | -0.099 | -0.155, -0.044 | <0.001 |
| Maternal education: complete secondary degree | -0.01 | -0.046, 0.027 | 0.598 |  | -0.019 | -0.067, 0.028 | 0.43 |
| Age in years | 0 | -0.014, 0.014 | 0.995 |  | -0.005 | -0.023, 0.014 | 0.616 |
| N | 7,285 |  |  |  | 7,285 |  |  |
| R2 | 0.155 |  |  |  | 0.164 |  |  |
| Note: CI = Confidence Interval; *, adjusted for false discovery rate with Benjamini-Hochberg method | | | | | | | |

| **Table S56 - Urban population regression analysis: poor quality of maternal-child relationship (get along in Brazil, closeness in UK)** | | | | | | | |
| --- | --- | --- | --- | --- | --- | --- | --- |
|  | **Internalising** | | |  | **Externalising** | | |
| **Characteristic** | **Beta** | **95% CI** | **p-value*** |  | **Beta** | **95% CI** | **p-value*** |
| Poor quality of maternal-child relationship | 0.035 | -0.008, 0.078 | 0.232 |  | 0.011 | -0.045, 0.068 | 0.868 |
| Poor quality of maternal-child relationship*Study (MCS:BHRCS) | 0.016 | -0.037, 0.069 | 0.668 |  | 0.069 | 0.000, 0.139 | 0.217 |
| **Covariates** |  |  |  |  |  |  |  |
| Study: MCS | -0.026 | -0.080, 0.029 | 0.359 |  | -0.548 | -0.620, -0.477 | <0.001 |
| SDQ factor score: Parent-report | 0.251 | 0.229, 0.274 | <0.001 |  | 0.438 | 0.410, 0.466 | <0.001 |
| Sex: female | 0.453 | 0.417, 0.488 | <0.001 |  | 0.073 | 0.027, 0.119 | 0.002 |
| Ethnicity: minority | -0.11 | -0.153, -0.067 | <0.001 |  | -0.098 | -0.155, -0.042 | <0.001 |
| Maternal education: complete secondary degree | -0.022 | -0.059, 0.014 | 0.235 |  | -0.033 | -0.081, 0.014 | 0.172 |
| Age in years | -0.002 | -0.017, 0.012 | 0.764 |  | -0.005 | -0.024, 0.014 | 0.581 |
| N | 7,163 |  |  |  | 7,163 |  |  |
| R2 | 0.154 |  |  |  | 0.164 |  |  |
| Note: CI = Confidence Interval; *, adjusted for false discovery rate with Benjamini-Hochberg method | | | | | | | |

| **Table S57 - Urban population regression analysis: maternal marital status** | | | | | | | |
| --- | --- | --- | --- | --- | --- | --- | --- |
|  | **Internalising** | | |  | **Externalising** | | |
| **Characteristic** | **Beta** | **95% CI** | **p-value*** |  | **Beta** | **95% CI** | **p-value*** |
| Separated and living with someone else | 0.148 | 0.067, 0.229 | 0.002 |  | 0.098 | -0.008, 0.203 | 0.252 |
| Single | 0.094 | 0.016, 0.172 | 0.062 |  | 0.048 | -0.054, 0.150 | 0.539 |
| Separated/divorced | 0.108 | 0.025, 0.192 | 0.045 |  | 0.161 | 0.052, 0.270 | 0.028 |
| Widow | 0.089 | -0.078, 0.257 | 0.493 |  | 0.116 | -0.103, 0.335 | 0.528 |
| Separated and living with someone else*Study (MCS:BHRCS) | -0.077 | -0.195, 0.041 | 0.404 |  | -0.075 | -0.229, 0.079 | 0.536 |
| Single*Study (MCS:BHRCS) | -0.005 | -0.106, 0.097 | 0.929 |  | 0.087 | -0.045, 0.219 | 0.417 |
| Separated/divorced*Study (MCS:BHRCS) | -0.035 | -0.140, 0.070 | 0.645 |  | 0.02 | -0.118, 0.157 | 0.868 |
| Widow*Study (MCS:BHRCS) | -0.161 | -0.443, 0.122 | 0.493 |  | -0.27 | -0.638, 0.099 | 0.37 |
| **Covariates** |  |  |  |  |  |  |  |
| Study: MCS | 0.009 | -0.045, 0.062 | 0.755 |  | -0.504 | -0.574, -0.435 | <0.001 |
| SDQ factor score: Parent-report | 0.25 | 0.228, 0.272 | <0.001 |  | 0.438 | 0.411, 0.466 | <0.001 |
| Sex: female | 0.452 | 0.417, 0.488 | <0.001 |  | 0.07 | 0.024, 0.117 | 0.003 |
| Ethnicity: minority | -0.111 | -0.154, -0.068 | <0.001 |  | -0.1 | -0.156, -0.044 | <0.001 |
| Maternal education: complete secondary degree | -0.012 | -0.048, 0.025 | 0.53 |  | -0.016 | -0.064, 0.032 | 0.506 |
| Age in years | -0.001 | -0.015, 0.013 | 0.89 |  | -0.006 | -0.025, 0.013 | 0.53 |
| N | 7,280 |  |  |  | 7,280 |  |  |
| R2 | 0.156 |  |  |  | 0.166 |  |  |
| Note: CI = Confidence Interval; *, adjusted for false discovery rate with Benjamini-Hochberg method | | | | | | | |

| **Table S58 - Urban population regression analysis: paternal death** | | | | | | | |
| --- | --- | --- | --- | --- | --- | --- | --- |
|  | **Internalising** | | |  | **Externalising** | | |
| **Characteristic** | **Beta** | **95% CI** | **p-value*** |  | **Beta** | **95% CI** | **p-value*** |
| Paternal death | 0.007 | -0.121, 0.134 | 0.929 |  | 0.006 | -0.161, 0.173 | 0.943 |
| Paternal death*Study (MCS:BHRCS) | 0.048 | -0.231, 0.327 | 0.819 |  | 0.186 | -0.179, 0.551 | 0.528 |
| **Covariates** |  |  |  |  |  |  |  |
| Study: MCS | -0.018 | -0.060, 0.023 | 0.389 |  | -0.506 | -0.560, -0.452 | <0.001 |
| SDQ factor score: Parent-report | 0.256 | 0.234, 0.278 | <0.001 |  | 0.449 | 0.422, 0.477 | <0.001 |
| Sex: female | 0.454 | 0.418, 0.489 | <0.001 |  | 0.074 | 0.028, 0.120 | 0.002 |
| Ethnicity: minority | -0.111 | -0.154, -0.068 | <0.001 |  | -0.105 | -0.161, -0.050 | <0.001 |
| Maternal education: complete secondary degree | -0.02 | -0.056, 0.017 | 0.289 |  | -0.029 | -0.077, 0.018 | 0.226 |
| Age in years | -0.001 | -0.015, 0.014 | 0.931 |  | -0.005 | -0.023, 0.014 | 0.614 |
| N | 7,285 |  |  |  | 7,285 |  |  |
| R2 | 0.152 |  |  |  | 0.162 |  |  |
| Note: CI = Confidence Interval; *, adjusted for false discovery rate with Benjamini-Hochberg method | | | | | | | |

| **Table S59 - Urban population regression analysis: difficulty in getting along or keeping friends** | | | | | | | |
| --- | --- | --- | --- | --- | --- | --- | --- |
|  | **Internalising** | | |  | **Externalising** | | |
| **Characteristic** | **Beta** | **95% CI** | **p-value*** |  | **Beta** | **95% CI** | **p-value*** |
| Difficulty in getting along or keeping friends | 0.161 | 0.048, 0.275 | 0.026 |  | 0.082 | -0.065, 0.230 | 0.514 |
| Difficulty in getting along or keeping friends*Study (MCS:BHRCS) | -0.05 | -0.173, 0.072 | 0.631 |  | 0.131 | -0.029, 0.290 | 0.329 |
| **Covariates** |  |  |  |  |  |  |  |
| Study: MCS | -0.029 | -0.074, 0.017 | 0.218 |  | -0.571 | -0.630, -0.512 | <0.001 |
| SDQ factor score: Parent-report | 0.249 | 0.226, 0.272 | <0.001 |  | 0.43 | 0.402, 0.458 | <0.001 |
| Sex: female | 0.464 | 0.428, 0.500 | <0.001 |  | 0.084 | 0.037, 0.131 | <0.001 |
| Ethnicity: minority | -0.089 | -0.133, -0.045 | <0.001 |  | -0.081 | -0.137, -0.024 | 0.005 |
| Maternal education: complete secondary degree | -0.025 | -0.062, 0.012 | 0.192 |  | -0.021 | -0.070, 0.027 | 0.389 |
| Age in years | 0.002 | -0.012, 0.016 | 0.798 |  | -0.004 | -0.022, 0.015 | 0.681 |
| N | 6,961 |  |  |  | 6,961 |  |  |
| R2 | 0.161 |  |  |  | 0.167 |  |  |
| Note: CI = Confidence Interval; *, adjusted for false discovery rate with Benjamini-Hochberg method | | | | | | | |

| **Table S60 - Urban population regression analysis: done bullying** | | | | | | | |
| --- | --- | --- | --- | --- | --- | --- | --- |
|  | **Internalising** | | |  | **Externalising** | | |
| **Characteristic** | **Beta** | **95% CI** | **p-value*** |  | **Beta** | **95% CI** | **p-value*** |
| Have you ever done bullying | 0.113 | 0.025, 0.202 | 0.045 |  | 0.164 | 0.049, 0.279 | 0.032 |
| Have you ever done bullying*Study (MCS:BHRCS) | 0.094 | -0.008, 0.195 | 0.161 |  | 0.302 | 0.170, 0.433 | <0.001 |
| **Covariates** |  |  |  |  |  |  |  |
| Study: MCS | -0.069 | -0.115, -0.023 | 0.003 |  | -0.621 | -0.681, -0.561 | <0.001 |
| SDQ factor score: Parent-report | 0.254 | 0.232, 0.276 | <0.001 |  | 0.427 | 0.400, 0.455 | <0.001 |
| Sex: female | 0.479 | 0.443, 0.515 | <0.001 |  | 0.109 | 0.062, 0.155 | <0.001 |
| Ethnicity: minority | -0.113 | -0.156, -0.069 | <0.001 |  | -0.102 | -0.158, -0.045 | <0.001 |
| Maternal education: complete secondary degree | -0.02 | -0.057, 0.016 | 0.274 |  | -0.03 | -0.078, 0.018 | 0.218 |
| Age in years | -0.002 | -0.017, 0.013 | 0.831 |  | -0.011 | -0.030, 0.009 | 0.281 |
| N | 7,005 |  |  |  | 7,005 |  |  |
| R2 | 0.166 |  |  |  | 0.184 |  |  |
| Note: CI = Confidence Interval; *, adjusted for false discovery rate with Benjamini-Hochberg method | | | | | | | |

| **Table S61 - Urban population regression analysis: been bullied** | | | | | | | |
| --- | --- | --- | --- | --- | --- | --- | --- |
|  | **Internalising** | | |  | **Externalising** | | |
| **Characteristic** | **Beta** | **95% CI** | **p-value*** |  | **Beta** | **95% CI** | **p-value*** |
| Have you ever been bullied | 0.211 | 0.148, 0.274 | <0.001 |  | 0.163 | 0.079, 0.246 | 0.002 |
| Have you ever been bullied*Study (MCS:BHRCS) | 0.153 | 0.076, 0.230 | 0.001 |  | 0.194 | 0.092, 0.296 | 0.002 |
| **Covariates** |  |  |  |  |  |  |  |
| Study: MCS | -0.136 | -0.186, -0.086 | <0.001 |  | -0.632 | -0.699, -0.566 | <0.001 |
| SDQ factor score: Parent-report | 0.239 | 0.216, 0.261 | <0.001 |  | 0.434 | 0.406, 0.462 | <0.001 |
| Sex: female | 0.465 | 0.430, 0.500 | <0.001 |  | 0.076 | 0.029, 0.122 | 0.001 |
| Ethnicity: minority | -0.089 | -0.132, -0.046 | <0.001 |  | -0.078 | -0.135, -0.021 | 0.007 |
| Maternal education: complete secondary degree | -0.029 | -0.065, 0.007 | 0.116 |  | -0.029 | -0.077, 0.019 | 0.234 |
| Age in years | 0.001 | -0.014, 0.015 | 0.918 |  | -0.007 | -0.026, 0.012 | 0.477 |
| N | 7,005 |  |  |  | 7,005 |  |  |
| R2 | 0.191 |  |  |  | 0.179 |  |  |
| Note: CI = Confidence Interval; *, adjusted for false discovery rate with Benjamini-Hochberg method | | | | | | | |

| **Table S62 - Urban population regression analysis: has at least one good friend, parent-report** | | | | | | | |
| --- | --- | --- | --- | --- | --- | --- | --- |
|  | **Internalising** | | |  | **Externalising** | | |
| **Characteristic** | **Beta** | **95% CI** | **p-value*** |  | **Beta** | **95% CI** | **p-value*** |
| The child does not have at least one good friend | 0.034 | -0.060, 0.128 | 0.645 |  | -0.03 | -0.153, 0.093 | 0.863 |
| The child does not have at least one good friend*Study (MCS:BHRCS) | 0.008 | -0.148, 0.163 | 0.929 |  | 0.019 | -0.185, 0.222 | 0.919 |
| **Covariates** |  |  |  |  |  |  |  |
| Study: MCS | -0.018 | -0.060, 0.024 | 0.409 |  | -0.508 | -0.564, -0.453 | <0.001 |
| SDQ factor score: Parent-report | 0.252 | 0.229, 0.275 | <0.001 |  | 0.449 | 0.421, 0.477 | <0.001 |
| Sex: female | 0.457 | 0.422, 0.492 | <0.001 |  | 0.075 | 0.029, 0.121 | 0.002 |
| Ethnicity: minority | -0.117 | -0.160, -0.074 | <0.001 |  | -0.108 | -0.164, -0.052 | <0.001 |
| Maternal education: complete secondary degree | -0.02 | -0.056, 0.016 | 0.271 |  | -0.032 | -0.080, 0.015 | 0.183 |
| Age in years | -0.001 | -0.015, 0.014 | 0.931 |  | -0.005 | -0.023, 0.014 | 0.622 |
| N | 7,251 |  |  |  | 7,251 |  |  |
| R2 | 0.153 |  |  |  | 0.161 |  |  |
| Note: CI = Confidence Interval; *, adjusted for false discovery rate with Benjamini-Hochberg method | | | | | | | |

| **Table S63 - Urban population regression analysis: has at least one good friend, self-report** | | | | | | | |
| --- | --- | --- | --- | --- | --- | --- | --- |
|  | **Internalising** | | |  | **Externalising** | | |
| **Characteristic** | **Beta** | **95% CI** | **p-value*** |  | **Beta** | **95% CI** | **p-value*** |
| I do not have at least one good friend | 0.032 | -0.087, 0.152 | 0.688 |  | 0.042 | -0.114, 0.199 | 0.853 |
| I do not have at least one good friend*Study (MCS:BHRCS) | 0.062 | -0.116, 0.240 | 0.645 |  | 0.01 | -0.222, 0.243 | 0.943 |
| **Covariates** |  |  |  |  |  |  |  |
| Study: MCS | -0.002 | -0.045, 0.041 | 0.925 |  | -0.493 | -0.549, -0.436 | <0.001 |
| SDQ factor score: Parent-report | 0.255 | 0.232, 0.277 | <0.001 |  | 0.449 | 0.421, 0.477 | <0.001 |
| Sex: female | 0.458 | 0.422, 0.494 | <0.001 |  | 0.073 | 0.026, 0.119 | 0.002 |
| Ethnicity: minority | -0.108 | -0.152, -0.065 | <0.001 |  | -0.11 | -0.167, -0.054 | <0.001 |
| Maternal education: complete secondary degree | -0.01 | -0.047, 0.026 | 0.585 |  | -0.021 | -0.069, 0.027 | 0.386 |
| Age in years | 0.002 | -0.013, 0.016 | 0.833 |  | -0.003 | -0.022, 0.017 | 0.797 |
| N | 7,094 |  |  |  | 7,094 |  |  |
| R2 | 0.153 |  |  |  | 0.158 |  |  |
| Note: CI = Confidence Interval; *, adjusted for false discovery rate with Benjamini-Hochberg method | | | | | | | |

| **Table S64 - Urban population regression analysis: moved to a different house or city since last interview** | | | | | | | |
| --- | --- | --- | --- | --- | --- | --- | --- |
|  | **Internalising** | | |  | **Externalising** | | |
| **Characteristic** | **Beta** | **95% CI** | **p-value*** |  | **Beta** | **95% CI** | **p-value*** |
| Moved to a different house or city since last interview | 0.071 | 0.006, 0.136 | 0.1 |  | 0.048 | -0.037, 0.133 | 0.514 |
| Moved to a different house or city since last interview*Study (MCS:BHRCS) | -0.03 | -0.120, 0.060 | 0.645 |  | 0.106 | -0.011, 0.224 | 0.252 |
| **Covariates** |  |  |  |  |  |  |  |
| Study: MCS | -0.007 | -0.052, 0.038 | 0.758 |  | -0.517 | -0.576, -0.458 | <0.001 |
| SDQ factor score: Parent-report | 0.254 | 0.232, 0.276 | <0.001 |  | 0.445 | 0.418, 0.473 | <0.001 |
| Sex: female | 0.454 | 0.419, 0.489 | <0.001 |  | 0.073 | 0.027, 0.119 | 0.002 |
| Ethnicity: minority | -0.109 | -0.152, -0.067 | <0.001 |  | -0.103 | -0.158, -0.047 | <0.001 |
| Maternal education: complete secondary degree | -0.021 | -0.057, 0.015 | 0.255 |  | -0.027 | -0.074, 0.021 | 0.269 |
| Age in years | 0.001 | -0.014, 0.015 | 0.907 |  | -0.004 | -0.023, 0.015 | 0.676 |
| N | 7,285 |  |  |  | 7,285 |  |  |
| R2 | 0.153 |  |  |  | 0.164 |  |  |
| Note: CI = Confidence Interval; *, adjusted for false discovery rate with Benjamini-Hochberg method | | | | | | | |

## P-Values pre and post adjustments

We applied the Benjamini-Hochberg method to account for multiple testing biases. An overview of all p-values pre and post correction for each regression is shown below. In the last column we indicate where p-values have changed in terms of their significance level (p<0.05) after correcting for multiple testing.

**Table S65: Overview of p-Values pre and post adjustments**

| **Predictor** | **Regression type** | **p-values** | **Adjusted p-values** | **Significance change** |
| --- | --- | --- | --- | --- |
| Number of people living in household | Main analysis (main+interaction) | 0.019 | 0.052 | Yes |
| Number of people living in household (by study; MCS) | Main analysis (main+interaction) | 0 | 0.01 | No |
| Siblings living in household | Main analysis (main+interaction) | 0.126 | 0.237 | No |
| Siblings living in household (by study; MCS) | Main analysis (main+interaction) | 0.184 | 0.307 | No |
| Halfsiblings living in household | Main analysis (main+interaction) | 0 | 0 | No |
| Halfsiblings living in household (by study; MCS) | Main analysis (main+interaction) | 0.666 | 0.799 | No |
| Poor quality of maternal-child relationship (get along in Brazil, closeness in UK) | Main analysis (main+interaction) | 0.031 | 0.078 | Yes |
| Poor quality of maternal-child relationship (get along in Brazil, closeness in UK) by study; MCS | Main analysis (main+interaction) | 0.847 | 0.941 | No |
| Maternal marital status: Separated and living with someone else | Main analysis (main+interaction) | 0 | 0 | No |
| Maternal marital status: Single | Main analysis (main+interaction) | 0.001 | 0.003 | No |
| Maternal marital status: Separated/divorced | Main analysis (main+interaction) | 0 | 0.001 | No |
| Maternal marital status: Widow | Main analysis (main+interaction) | 0.141 | 0.249 | No |
| Maternal marital status: Separated and living with someone else (by study; MCS) | Main analysis (main+interaction) | 0.096 | 0.205 | No |
| Maternal marital status: Single (by study; MCS) | Main analysis (main+interaction) | 0.46 | 0.601 | No |
| Maternal marital status: Separated/divorced (by study; MCS) | Main analysis (main+interaction) | 0.726 | 0.838 | No |
| Maternal marital status: Widow (by study; MCS) | Main analysis (main+interaction) | 0.529 | 0.661 | No |
| Paternal death | Main analysis (main+interaction) | 0.942 | 0.942 | No |
| Paternal death (by study; MCS) | Main analysis (main+interaction) | 0.912 | 0.942 | No |
| Difficulty in getting along or keeping friends | Main analysis (main+interaction) | 0 | 0 | No |
| Difficulty in getting along or keeping friends (by study; MCS) | Main analysis (main+interaction) | 0.26 | 0.411 | No |
| Have you ever done bullying | Main analysis (main+interaction) | 0 | 0.001 | No |
| Have you ever done bullying (by study; MCS) | Main analysis (main+interaction) | 0.034 | 0.079 | Yes |
| Have you ever been bullied | Main analysis (main+interaction) | 0 | 0 | No |
| Have you ever been bullied (by study; MCS) | Main analysis (main+interaction) | 0 | 0 | No |
| The child does not have at least one good | Main analysis (main+interaction) | 0.336 | 0.48 | No |
| The child does not have at least one good (by study; MCS) | Main analysis (main+interaction) | 0.916 | 0.942 | No |
| I do not have at least one good friend | Main analysis (main+interaction) | 0.424 | 0.578 | No |
| I do not have at least one good friend (by study; MCS) | Main analysis (main+interaction) | 0.289 | 0.433 | No |
| Moved to a different house or city since last interview | Main analysis (main+interaction) | 0 | 0.001 | No |
| Moved to a different house or city since last interview (by study; MCS) | Main analysis (main+interaction) | 0.112 | 0.224 | No |
| Number of people living in household | BHRCS only | 0.329 | 0.501 | No |
| Siblings living in household | BHRCS only | 0.406 | 0.553 | No |
| Halfsiblings living in household | BHRCS only | 0.016 | 0.086 | Yes |
| Poor quality of maternal-child relationship (get along in Brazil, closeness in UK) | BHRCS only | 0.334 | 0.501 | No |
| Maternal marital status: Separated and living with someone else | BHRCS only | 0.017 | 0.086 | Yes |
| Maternal marital status: Single | BHRCS only | 0.13 | 0.243 | No |
| Maternal marital status: Separated/divorced | BHRCS only | 0.099 | 0.213 | No |
| Maternal marital status: Widow | BHRCS only | 0.506 | 0.633 | No |
| Paternal death | BHRCS only | 0.908 | 0.908 | No |
| Difficulty in getting along or keeping friends | BHRCS only | 0.04 | 0.148 | Yes |
| Have you ever done bullying | BHRCS only | 0.069 | 0.173 | No |
| Have you ever been bullied | BHRCS only | 0 | 0 | No |
| The child does not have at least one good | BHRCS only | 0.612 | 0.706 | No |
| I do not have at least one good friend | BHRCS only | 0.708 | 0.758 | No |
| Moved to a different house or city since last interview | BHRCS only | 0.055 | 0.166 | No |
| Number of people living in household | MCS only | 0.001 | 0.002 | No |
| Siblings living in household | MCS only | 0 | 0.001 | No |
| Halfsiblings living in household | MCS only | 0.009 | 0.015 | No |
| Poor quality of maternal-child relationship (get along in Brazil, closeness in UK) | MCS only | 0 | 0.001 | No |
| Maternal marital status: Separated and living with someone else | MCS only | 0.061 | 0.092 | No |
| Maternal marital status: Single | MCS only | 0.002 | 0.004 | No |
| Maternal marital status: Separated/divorced | MCS only | 0.006 | 0.01 | No |
| Maternal marital status: Widow | MCS only | 0.585 | 0.585 | No |
| Paternal death | MCS only | 0.572 | 0.585 | No |
| Difficulty in getting along or keeping friends | MCS only | 0 | 0 | No |
| Have you ever done bullying | MCS only | 0 | 0 | No |
| Have you ever been bullied | MCS only | 0 | 0 | No |
| The child does not have at least one good friend | MCS only | 0.541 | 0.585 | No |
| I do not have at least one good friend | MCS only | 0.151 | 0.189 | No |
| Moved to a different house or city since last interview | MCS only | 0.077 | 0.105 | No |
| Number of people living in household | Unajusted | 0.001 | 0.003 | No |
| Number of people living in household (by study; MCS) | Unajusted | 0 | 0 | No |
| Siblings living in household | Unajusted | 0.816 | 0.92 | No |
| Siblings living in household (by study; MCS) | Unajusted | 0.001 | 0.002 | No |
| Halfsiblings living in household | Unajusted | 0 | 0 | No |
| Halfsiblings living in household (by study; MCS) | Unajusted | 0.851 | 0.92 | No |
| Poor quality of maternal-child relationship (get along in Brazil, closeness in UK) | Unajusted | 0 | 0 | No |
| Poor quality of maternal-child relationship (get along in Brazil, closeness in UK) by study; MCS | Unajusted | 0.343 | 0.468 | No |
| Maternal marital status: Separated and living with someone else | Unajusted | 0 | 0 | No |
| Maternal marital status: Single | Unajusted | 0 | 0 | No |
| Maternal marital status: Separated/divorced | Unajusted | 0 | 0 | No |
| Maternal marital status: Widow | Unajusted | 0.192 | 0.321 | No |
| Maternal marital status: Separated and living with someone else (by study; MCS) | Unajusted | 0.318 | 0.454 | No |
| Maternal marital status: Single (by study; MCS) | Unajusted | 0.065 | 0.115 | No |
| Maternal marital status: Separated/divorced (by study; MCS) | Unajusted | 0.495 | 0.646 | No |
| Maternal marital status: Widow (by study; MCS) | Unajusted | 0.859 | 0.92 | No |
| Paternal death | Unajusted | 0.671 | 0.805 | No |
| Paternal death (by study; MCS) | Unajusted | 0.918 | 0.95 | No |
| Difficulty in getting along or keeping friends | Unajusted | 0 | 0 | No |
| Difficulty in getting along or keeping friends (by study; MCS) | Unajusted | 0.001 | 0.002 | No |
| Have you ever done bullying | Unajusted | 0.002 | 0.005 | No |
| Have you ever done bullying (by study; MCS) | Unajusted | 0.315 | 0.454 | No |
| Have you ever been bullied | Unajusted | 0 | 0 | No |
| Have you ever been bullied (by study; MCS) | Unajusted | 0 | 0 | No |
| The child does not have at least one good | Unajusted | 0 | 0 | No |
| The child does not have at least one good (by study; MCS) | Unajusted | 0.664 | 0.805 | No |
| I do not have at least one good friend | Unajusted | 0.275 | 0.435 | No |
| I do not have at least one good friend (by study; MCS) | Unajusted | 0.048 | 0.089 | Yes |
| Moved to a different house or city since last interview | Unajusted | 0.001 | 0.003 | No |
| Moved to a different house or city since last interview (by study; MCS) | Unajusted | 0.957 | 0.957 | No |
| Number of people living in household | Urban only | 0,06 | 0.151 | No |
| Number of people living in household (by study; MCS) | Urban only | 0 | 0.001 | No |
| Siblings living in household | Urban only | 0.415 | 0.631 | No |
| Siblings living in household (by study; MCS) | Urban only | 0.06 | 0.151 | No |
| Halfsiblings living in household | Urban only | 0 | 0.001 | No |
| Halfsiblings living in household (by study; MCS) | Urban only | 0.285 | 0.493 | No |
| Poor quality of maternal-child relationship (get along in Brazil, closeness in UK) | Urban only | 0.108 | 0.232 | No |
| Poor quality of maternal-child relationship (get along in Brazil, closeness in UK) by study; MCS | Urban only | 0.557 | 0.668 | No |
| Maternal marital status: Separated and living with someone else | Urban only | 0 | 0.002 | No |
| Maternal marital status: Single | Urban only | 0.019 | 0.062 | Yes |
| Maternal marital status: Separated/divorced | Urban only | 0.011 | 0.045 | No |
| Maternal marital status: Widow | Urban only | 0.296 | 0.493 | No |
| Maternal marital status: Separated and living with someone else (by study; MCS) | Urban only | 0.202 | 0.404 | No |
| Maternal marital status: Single (by study; MCS) | Urban only | 0.929 | 0.929 | No |
| Maternal marital status: Separated/divorced (by study; MCS) | Urban only | 0.514 | 0.645 | No |
| Maternal marital status: Widow (by study; MCS) | Urban only | 0.264 | 0.493 | No |
| Paternal death | Urban only | 0.92 | 0.929 | No |
| Paternal death (by study; MCS) | Urban only | 0.737 | 0.819 | No |
| Difficulty in getting along or keeping friends | Urban only | 0.005 | 0.026 | No |
| Difficulty in getting along or keeping friends (by study; MCS) | Urban only | 0.421 | 0.631 | No |
| Have you ever done bullying | Urban only | 0.012 | 0.045 | No |
| Have you ever done bullying (by study; MCS) | Urban only | 0.07 | 0.161 | No |
| Have you ever been bullied | Urban only | 0 | 0 | No |
| Have you ever been bullied (by study; MCS) | Urban only | 0 | 0.001 | No |
| The child does not have at least one good | Urban only | 0.477 | 0.645 | No |
| The child does not have at least one good (by study; MCS) | Urban only | 0.922 | 0.929 | No |
| I do not have at least one good friend | Urban only | 0.596 | 0.688 | No |
| I do not have at least one good friend (by study; MCS) | Urban only | 0.497 | 0.645 | No |
| Moved to a different house or city since last interview | Urban only | 0.033 | 0.1 | Yes |
| Moved to a different house or city since last interview (by study; MCS) | Urban only | 0.516 | 0.645 | No |

##

## Lived Experience Workshop

Lived experience workshop agenda

- **Introduction**
- **Ice-breaker activities**
- **Part 1: Young Peoples’ Social Connections**
  - (i) Examples of what positive social interactions are for young people
  - What are the benefits to young people of having social interactions? How do they make you feel?
- **Part 2: Barriers to Social Interactions and the Impact on Young People**
  - Impact of Barriers to Social Interactions
  - Identify the key contributing factors that can impact on social interactions

### **Examples Part 1 activities:**


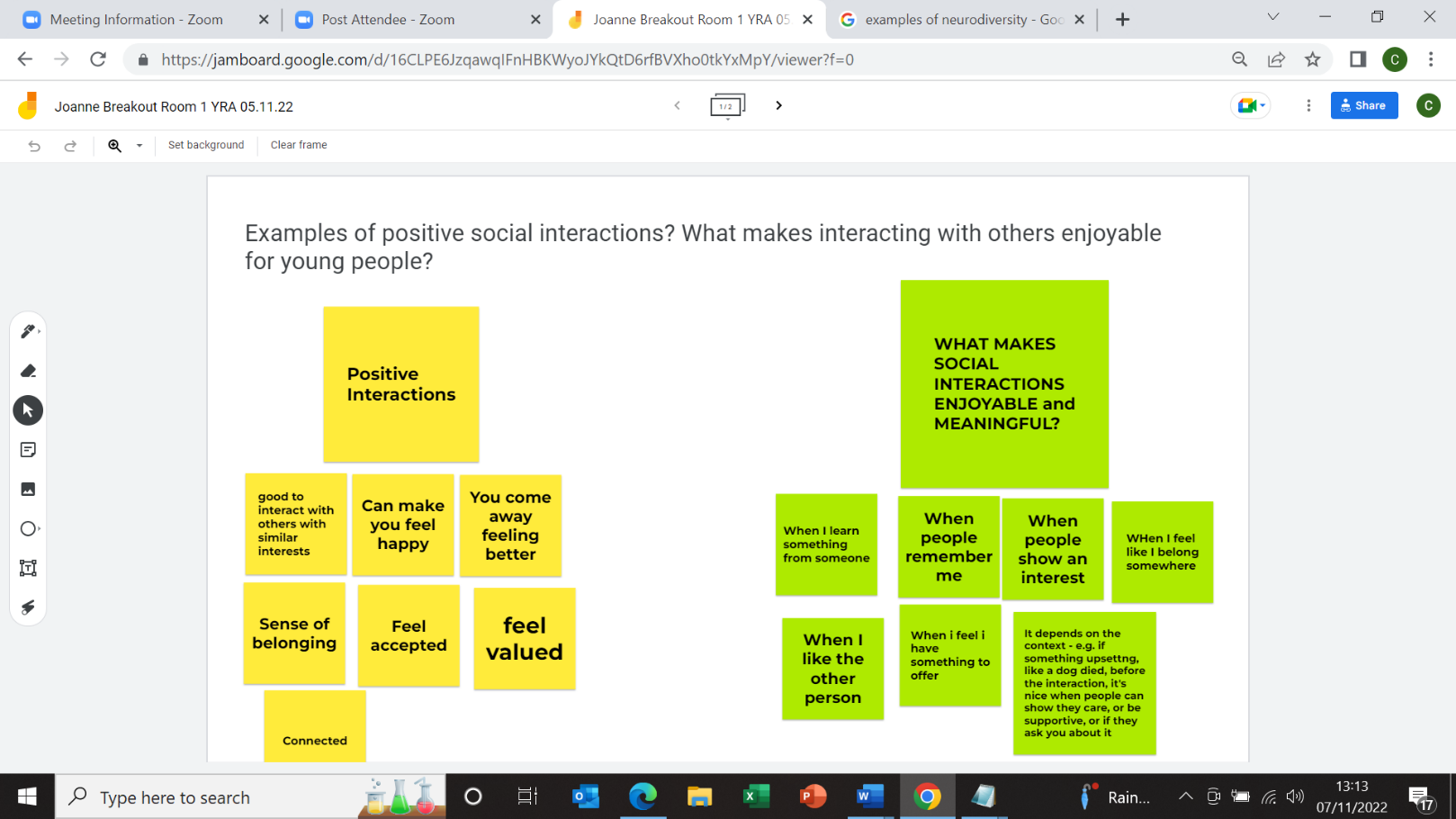
Figure S3. Jam board completed by young advisors

### **Examples Part 2 activities**

Figure S4. Jam board completed by young advisors


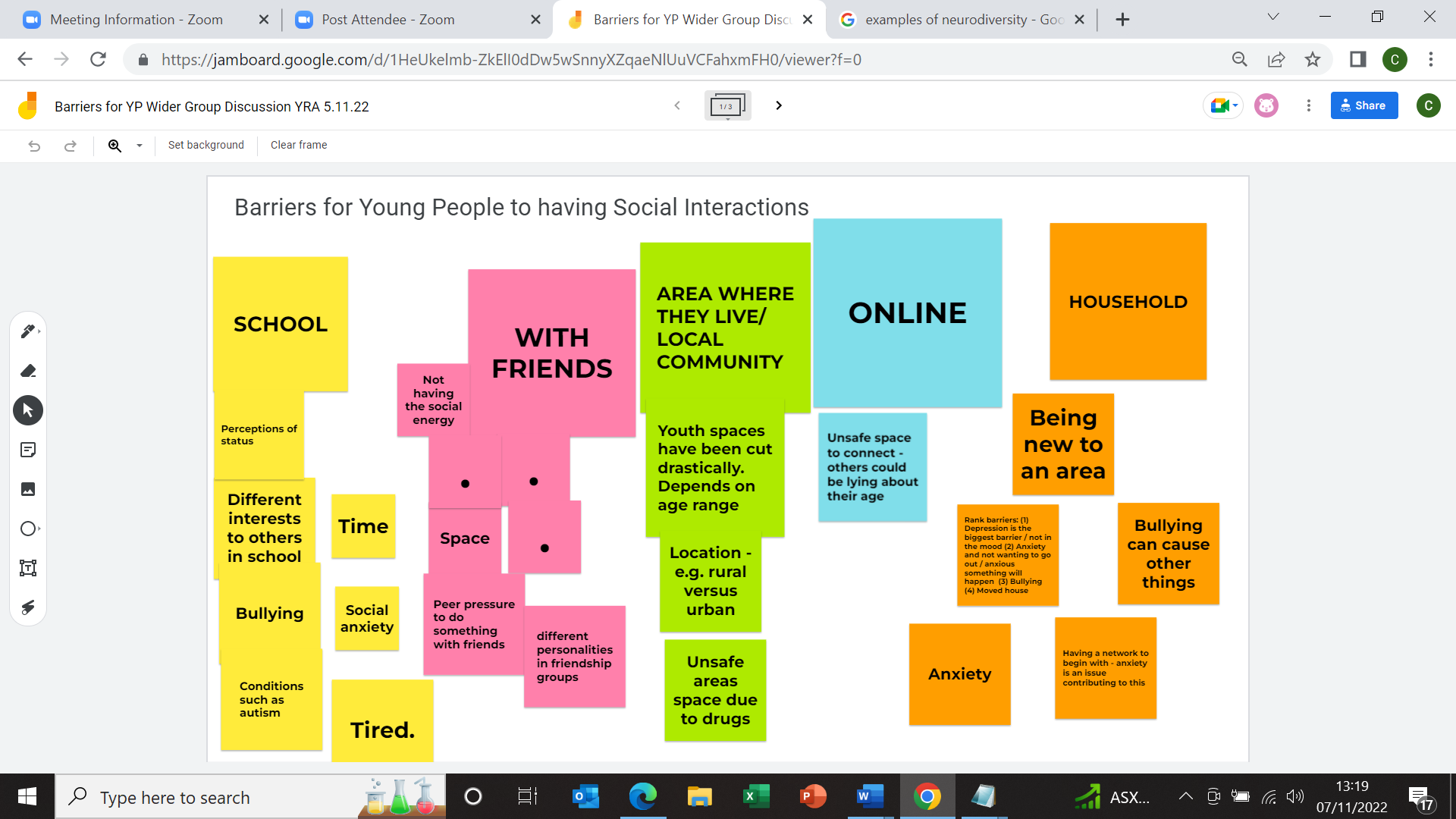

Supplement: Supplementary file 1 — Figure S1. Sensitivity analysis for internalising symptoms. Figure S2. Sensitivity analysis for externalising symptoms. Figure S3. Jam board completed by young advisors. Figure S4. Jam board completed by young advisors. Table S1. Overview of harmonised items. Table S2. Confirmatory factor analysis scalar‐invariant model of SDQ domains across different informants. Table S3. Measurement invariance of parent‐report SDQ across study type. Table S4. Measurement invariance of self‐report SDQ across study type. Tables S5–S16. Pooled regression analysis: respective social connection factor. Tables S17–S28. BHRCS regression analysis: respective social connection factor. Tables S29–S40. MCS regression analysis: respective social connection factor. Tables S41–S52. Unadjusted regression analysis: respective social connection factor. Tables S53–S64. Urban population regression analysis: respective social connection factor. Table S65. Overview of p‐values pre‐ and post‐adjustments. [file JCPP-66-725-s001.docx]
